# Improved objective Bayesian estimator for a PLP model hierarchically represented subject to competing risks under minimal repair regime

**DOI:** 10.1371/journal.pone.0255944

**Published:** 2021-08-12

**Authors:** Francisco Louzada, José A. Cuminato, Oscar M. H. Rodriguez, Vera L. D. Tomazella, Paulo H. Ferreira, Pedro L. Ramos, Eder A. Milani, Gustavo Bochio, Ivan C. Perissini, Oilson A. Gonzatto Junior, Alex L. Mota, Luis F. A. Alegría, Danilo Colombo, Eduardo A. Perondi, André V. Wentz, Anselmo L. Silva Júnior, Dante A. C. Barone, Hugo F. L. Santos, Marcus V. C. Magalhães

**Affiliations:** 1 Institute of Mathematical and Computer Sciences (ICMC), University of São Paulo, São Carlos, SP, Brazil; 2 School of Engineering (EESC), University of São Paulo, São Carlos, SP, Brazil; 3 Department of Statistics (DEs), Federal University of São Carlos, São Carlos, SP, Brazil; 4 Department of Statistics (DEst), Federal University of Bahia, Salvador, BA, Brazil; 5 Pontificia Universidad Católica de Chile, Facultad de Matemáticas, Macul, Santiago, Chile; 6 Institute of Mathematics and Statistics (IME), Federal University of Goiás, Goiânia, GO, Brazil; 7 Leopoldo Américo Miguez de Mello Research and Development Center (CENPES—Petrobras), Rio de Janeiro, RJ, Brazil; 8 Department of Mechanical Engineering, Federal University of Rio Grande do Sul, Porto Alegre, RS, Brazil; 9 National Service of Industrial Training (SENAI), São Leopoldo, RS, Brazil; 10 National Service of Industrial Training (SENAI), Florianópolis, SC, Brazil; 11 Institute of Informatics (Inf), Federal University of Rio Grande do Sul, Porto Alegre, RS, Brazil; Universidad Rey Juan Carlos, SPAIN

## Abstract

In this paper, we propose a hierarchical statistical model for a single repairable system subject to several failure modes (competing risks). The paper describes how complex engineered systems may be modelled hierarchically by use of Bayesian methods. It is also assumed that repairs are minimal and each failure mode has a power-law intensity. Our proposed model generalizes another one already presented in the literature and continues the study initiated by us in another published paper. Some properties of the new model are discussed. We conduct statistical inference under an objective Bayesian framework. A simulation study is carried out to investigate the efficiency of the proposed methods. Finally, our methodology is illustrated by two practical situations currently addressed in a project under development arising from a partnership between Petrobras and six research institutes.

## 1 Introduction

The challenges in the production of offshore oil wells have been increasing over time, either due to the increase in technical difficulties because of the greater complexity of the areas to be explored, or due to improvements in the rules of the regulatory bodies in order to increase safety. There are two key pillars that should guide an oil well project: safety and productivity.

**Fig 1:**
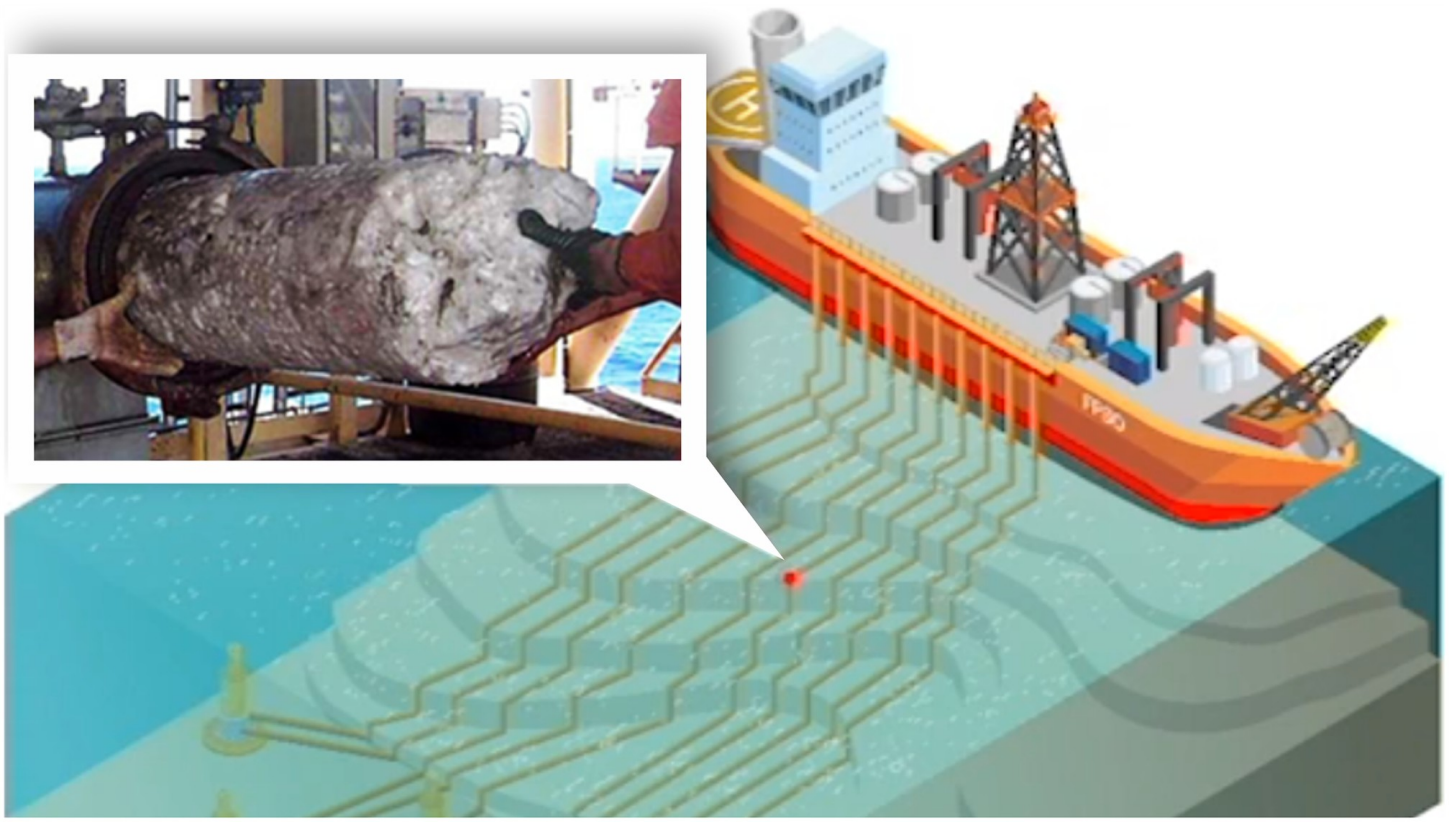
Hydrates and paraffins. The most common duct obstruction causes.

Petroleum industry loses billions of dollars yearly due to profit loss associated with production lines obstruction. Current flow assurance solutions are troublesome and cost hundreds of millions of dollars annually. Petrobras (abbreviation of *Petróleo Brasileiro S.A.*), which is Brazil’s largest oil and gas producer, has invested in technological innovation projects in order to minimize these losses and increase oil and gas production. Annelida is one of these Petrobras’ innovation projects, which has been developed in partnership with the main Brazil’s research centers. It regards an in-pipe robot that will be used at a near future to remove hydrates and paraffins that form in pipelines and can cause problems in oil and gas flow (see Fig 1). Several stages of the Annelida project have already been completed and many others are underway, generating important results for the development and improvement of its bases. Given the innovative nature of the project, the reliability modeling of the product has been one of the main objectives of the research centers.

**Fig 2:**
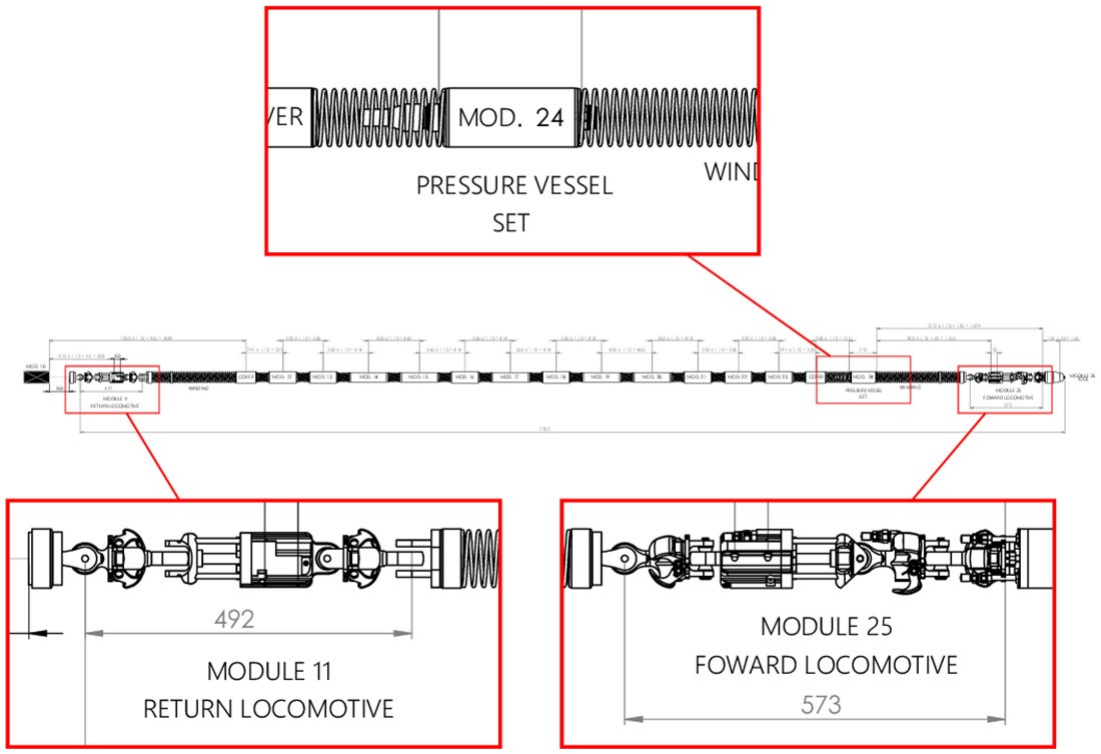
Annelida’s schematic diagram. The representation of one of the pressure vessel set (top), the others (modules 01 to 10) are not represented in this schematic, and traction systems (below).

In reliability engineering, it is well known that the reliability of a product can be assessed from the systems and subsystems that comprise it. Annelida is composed of several systems and subsystems each with well-defined objectives. Due to the high degree of criticality, in this work we consider the traction system of Annelida. In particular, we will study (that is, model the failure times of) the return and forward locomotives subsystem (modules 11 and 25, in Fig 2), as well as the pressure vessel subsystem (modules 1 to 10 and 24, one of which is represented in module 24, in Fig 2). A schematic of the studied systems is shown in Fig 2.

The locomotive is responsible for conducting the robot inside the pipe, and once hydrate formation is identified, the robot will work on its safe removal for the oil to flow again. On the other hand, the pressure vessel set is the basic structural module for all the electrical and electronic components of Annelida, which contains 11 of these subsystems. The vessels’ purpose is to withstand the forces, pressure and chemical conditions of the environment, safety containing and isolating the components in their interiors. The module also has the function to facilitate the heat exchange, allowing for suitable operational temperature of the electronic components.

## 2 Background and literature

The repeated occurrence of an event of interest in the same subject is frequent in many areas, such as manufacturing, software development, medicine, social sciences, risk analysis, among others. In reliability engineering, when studying a complex system, such as supercomputers, cars and airplanes, multiple defects or vulnerabilities in the product design, manufacture, operation, maintenance and handling can cause a number of unexpected failures [1]. Failure process models, in the context of repairable systems, are often described in terms of competing risks, or equivalently, a system with many components connected in series, such that the failure of a single component will result in a whole system failure. However, recently in engineering, the evaluation of repairable systems with multiple failure modes has drawn attention due to their potential applicability [2–5].

In a competing risks framework, a system fails due to the first occurrence of possible failure modes. In this context, we use a model for any individual component, whose failures occur due to one of the causal mechanisms and which each one acts independently on the system.

A system can be thought as the joint union of different subsystems which, in turn, can also be thought as unions of other more particular subsystems, to an arbitrary level of hierarchy. In a well-defined hierarchical structure, the functions and relationships between components of a system can be better understood, highlighting their importance to the system as a whole. This makes it possible to clearly define acceptable levels of damage for each part of the structure, and to delimit its impacts on the system when exposed to different sources of external variation [6].

In an industrial context, Langseth and Lindqvist [7] recorded the service times of a component spanning over 1,600 units of time. Each failure had its respective mode also recorded. In this case, the causes of failure were categorized into two main groups, each with its respective subcauses. In health care, for example, Tuli *et al.* [8] analyzed repeated shunt failures in children diagnosed with hydrocephalus; failures in this context are known to result from a variety of causes.

In complex systems with several hierarchical levels, redundancy can be implemented in any of the hierarchical levels. Finding the specific optimal configuration of a specific system is addressed by the reliability allocation problem. At the lowest level of the hierarchy, a unit can have different failure modes. Considering the modes separately might be of importance as either the consequences of the failures might be different or the maintenance actions that each failure mode triggers might be different. In general, the failure of any single component can be considered in a competing risks framework where every failure mode is competing against the others to make the component fail in that mode.

A repairable system is defined as a system which, after failing to perform one or more of its functions satisfactorily, can be restored to fully satisfactory performance by a method other than replacement of the entire system. Traditional studies on repairable systems focus on modeling failure times, using point process theory as the main tool. In the literature, it is commonly assumed that failures in a repairable system occur due to a Non-Homogeneous Poisson Process (NHPP) with the intensity described by a power law. The resulting method is generally referred to as the Power Law Process (PLP). The PLP is convenient in many ways, especially for its flexibility, easy implementation, and the interpretability of its parameters [9, 10].

Considering the fault-causing mechanisms known, it is also important to observe how to repair such failures, including preventive maintenance. In this context, the books of Crowder [11], Pintilie [12], among others, illustrate with some examples the need for considering the setting of competing risks in the application of reliability techniques (in industrial statistics) or survival analysis tools (in health sciences).

Under a Bayesian perspective, the inference of a problem is on the basis of the posterior distribution of the quantity of interest, which combines the information provided by the data with the available prior information. The elicitation of an appropriate prior distribution becomes the main task for Bayesian statisticians in practice. Subjective priors, which always depend on the experts’ belief, are not easy to derive in a limited time period. Therefore, given little prior information, we prefer to use objective (non-informative) priors to make inference.

An important objective prior distribution is the reference prior, introduced by Bernardo [13] and later refined by other authors [14–17]. The reference prior is minimally informative in a precise theoretical sense about information. The intent is to make information from data dominate *a priori* information, reflecting the vague nature of *a priori* knowledge. To obtain such prior, the expected Kullback-Leibler divergence between a prior distribution and a posterior distribution was maximized. The posterior distribution obtained using this prior has interesting properties, such as invariance and consistency in marginalization and sample properties [18]. Some recent reference priors were obtained for the Pareto [19], Poisson-exponential [20], extended exponential-geometric [21], inverse Weibull [22], generalized half-normal [23] and Lomax [24] distributions.

This paper aims to continue the study begun in Louzada *et al.* [25], which generalized the Somboonsavatdee and Sen’s model [26]. For this, we describe a statistical model for a repairable system hierarchically represented subject to competing risks under minimal repair regime with PLP intensity function. Working under an objective Bayesian framework, we consider reference and matching priors for the model parameters. The proposed methodology is applied to two real problems arising from the development of the robotic unit Annelida, as described previously. Since the robot is not ready yet, and real experimental data are difficult to obtain and use at the early stages of a complex technological innovation project like this, the failure time data analyzed here are synthetic ones generated using the limited but currently available information provided by the technical team’s FMEA (Failure Mode and Effects Analysis) and FTA (Fault Tree Analysis). Despite this, the proposed hierarchical statistical model and methods prove to be useful in studying the reliability of the several systems and subsystems that comprise the product.

The next sections of the paper are organized as follows. In Section 3, we introduce the proposed statistical model, which considers, in its basic assumptions, a hierarchically represented repairable system (with an arbitrary number of hierarchical levels) subject to competing risks in a minimal repair regime governed by a PLP intensity function. In Section 4, under the perspective of an objective Bayesian inference framework, we derive the maximum *a posteriori* probability (MAP) estimators for the parameters of the proposed model, correct the biases of such estimators and expose credibility intervals with closed forms. In Section 5, we evaluate the properties of the estimators through a simulation study. In Section 6, we use the proposed model (and methods) to assess the reliability of two subsystems of the robotic unit (pressure vessels and traction system) that motivated this research, in the light of currently available information. Finally, in Section 7 we draw some final remarks and suggestions for future research.

## 3 Model formulation

**Fig 3:**
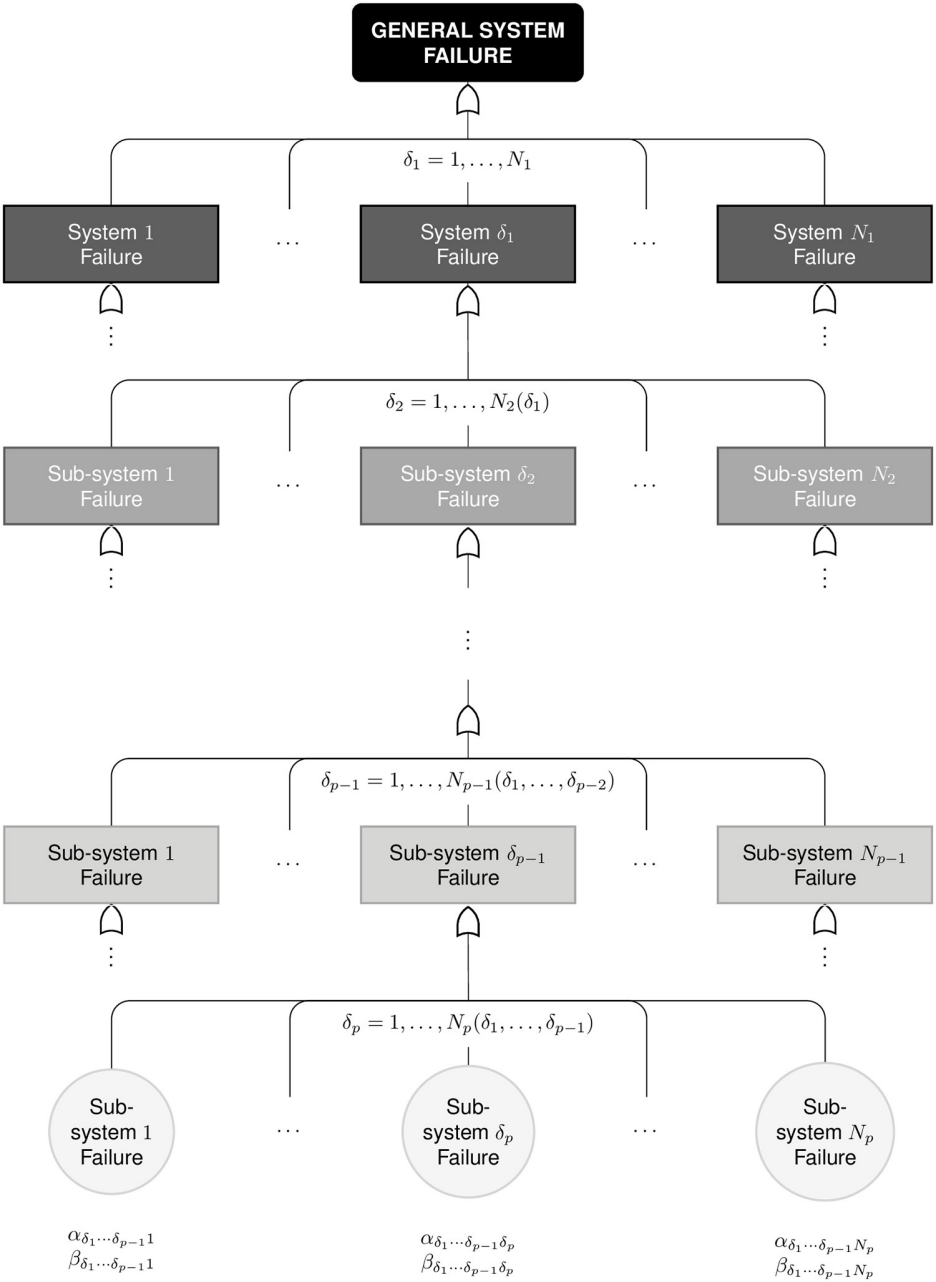
System composition. Fault tree analysis (FTA) of the general system with hierarchical failure modes (*p* levels of nesting).

In this section, we introduce the proposed statistical modeling for reliability data arising from a single repairable system subject to both minimal repairs and hierarchical competing risks, whose successive failures are assumed to be governed by a PLP. Our model can be regarded as a generalization of the Somboonsavatdee and Sen [26]’s model for the cases where there are two or more levels of hierarchy, that is, secondary, tertiary, quaternary and so on failure causes (or subsystems). The model proposed here is also an extension of the work by Louzada *et al.* [25]. This situation is illustrated in Fig 3, which depicts a fault tree. The general feature illustrated in this figure includes the composition of a system by multiple subsystems, and the composition of these subsystems by further subsystems and components.

In order to model these kinds of systems, we first assume that the failure probabilities of components in distinct branches of the fault tree are conditionally independent and that success of the systems requires successful functioning of all components.

Let us suppose a repairable system with *p* levels of hierarchy. Then, the hierarchical competing risks model’s data consist of the (*p* + 1)-tuples (*t*, *δ*_1_(*t*), *δ*_2_(*t*), …, *δ*_*p*_(*t*)), where *t* > 0 denotes the failure time, *δ*_1_(*t*) is the indicator of the leading failure cause (system) at the failure time *t*, and *δ*_*j*_(*t*) is the indicator of the subcause (subsystem) at hierarchical level *j* and at the failure time *t*, for *j* = 2, …, *p* (in what follows, we will suppress the explicit dependence of *δ*_*j*_ on failure time *t* for brevity).

Let *N*_*p*_(*δ*_*p*_) be the counting process associated with the system failure of type *p*. To consider the natural hierarchy of this model, the *δ*_2_ indicator, for example, refers to the cause in the second hierarchical level, which is nested with a specific cause of the first hierarchical level, represented by *δ*_1_. In this sense, we say that *δ*_1_ = 1, …, *N*_1_, that is, there are *N*_1_ primary causes of system failure. On the other hand, *δ*_2_ = 1, …, *N*_2_(*δ*_1_), that is, the number of causes *N*_2_(*δ*_1_) (denoted later simply by *N*_2_, for simplicity) closely depends on the primary cause *δ*_1_. This logic extends to the *p*-th cause, indicated by *δ*_*p*_ = 1, …, *N*_*p*_(*δ*_1_, …, *δ*_*p*−1_).

Our proposed model for failure data analysis can be formulated as follows. Let $N_{\delta_{1}\ldots \delta_{p}}\left(t\right)$ be the hierarchical (at level *p*) subsystem-specific counting process, which denotes the number of failures before time *t*. It is easy to demonstrate that, for the system level, the cumulative failure counter is $N\left(t\right)=\sum_{\delta_{1}=1}^{N_{1}}\cdots \sum_{\delta_{p}=1}^{N_{p}}N_{\delta_{1}\ldots \delta_{p}}\left(t\right)$. Then, assume that the failures from a hierarchical (at level *p*) subsystem follow a NHPP with the PLP intensity function given by

$$\begin{array}{cc} \lambda_{\delta_{1}\cdots \delta_{p}}\left(t\right) & =\lim_{\Delta t\to 0}\frac{P(\delta_{1}\left(t\right)=\delta_{1},\ldots ,\delta_{p}\left(t\right)=\delta_{p},N\left(t+\Delta t\right)-N\left(t\right)=1|N\left(s\right),0\leq s\leq t)}{\Delta t}\\ & =(\frac{\beta_{\delta_{1}\cdots \delta_{p}}}{\mu_{\delta_{1}\cdots \delta_{p}}}){\left(\frac{t}{\mu_{\delta_{1}\cdots \delta_{p}}}\right)}^{\beta_{\delta_{1}\cdots \delta_{p}}-1}, \end{array}$$

where $\mu_{\delta_{1}\ldots \delta_{p}}>0$ and $\beta_{\delta_{1}\ldots \delta_{p}}>0$ are, respectively, the scale and shape parameters. Or equivalently, $\mu_{\delta_{1}\ldots \delta_{p}}$ represents the time for which we expect to observe a single event, and $\beta_{\delta_{1}\ldots \delta_{p}}$ is the elasticity of the mean number of events with regard to time [27].

Thus, it follows that the overall intensity function at time *t* is given by

$$\begin{array}{cc} \lambda (t) & =\sum_{\delta_{1}=1}^{N_{1}}\cdots \sum_{\delta_{p}=1}^{N_{p}}\lambda_{\delta_{1}\cdots \delta_{p}}\left(t\right)\\ & =\sum_{\delta_{1}=1}^{N_{1}}\cdots \sum_{\delta_{p}=1}^{N_{p}}(\frac{\beta_{\delta_{1}\cdots \delta_{p}}}{\mu_{\delta_{1}\cdots \delta_{p}}}){\left(\frac{t}{\mu_{\delta_{1}\cdots \delta_{p}}}\right)}^{\beta_{\delta_{1}\cdots \delta_{p}}-1}, \end{array}$$ (1)

where *N*_*j*_ denotes the number of components in the (*δ*_1_, …, *δ*_*j*_)-th hierarchical subsystem, for *j* = 1, …, *p*.

Let us assume that *n* ≥ 1 failures have occurred in the time interval (0, *T*]. Then, the hierarchical (at level *p*) subsystem-specific cumulative intensity up to time *T* becomes

$$\begin{array}{c} \Lambda_{\delta_{1}\cdots \delta_{p}}\left(T\right)={\left(\frac{T}{\mu_{\delta_{1}\cdots \delta_{p}}}\right)}^{\beta_{\delta_{1}\cdots \delta_{p}}}. \end{array}$$

From Eq (3), it follows that

$$\begin{array}{cc} \Lambda (T) & =\sum_{\delta_{1}=1}^{N_{1}}\cdots \sum_{\delta_{p}=1}^{N_{p}}\Lambda_{\delta_{1}\cdots \delta_{p}}\left(T\right) \end{array}$$

is the overall cumulative intensity up to time *T*. Hence, we have that the overall reliability up to time *T* is

$$\begin{array}{cc} R(T) & =\exp\{-\Lambda (T)\}\\ & =\exp\{-\sum_{\delta_{1}=1}^{N_{1}}\cdots \sum_{\delta_{p}=1}^{N_{p}}\Lambda_{\delta_{1}\cdots \delta_{p}}\left(T\right)\}\\ & =\exp\{-\sum_{\delta_{1}=1}^{N_{1}}\cdots \sum_{\delta_{p}=1}^{N_{p}}{\left(\frac{T}{\mu_{\delta_{1}\cdots \delta_{p}}}\right)}^{\beta_{\delta_{1}\cdots \delta_{p}}}\}, \end{array}$$

while the hierarchical (at level *p*) subsystem-specific reliability up to time *T* is given by

$$\begin{array}{cc} R_{\delta_{1}\cdots \delta_{p}}\left(T\right) & =\exp\{-\Lambda_{\delta_{1}\cdots \delta_{p}}\left(T\right)\}=\exp\{-{\left(\frac{T}{\mu_{\delta_{1}\cdots \delta_{p}}}\right)}^{\beta_{\delta_{1}\cdots \delta_{p}}}\}. \end{array}$$

As suggested by Oliveira *et al.* [27], we will reparametrize our proposed model in terms of $\beta_{\delta_{1}\ldots \delta_{p}}$ and

$$\begin{array}{cc} \alpha_{\delta_{1}\cdots \delta_{p}} & =E\left[N_{\delta_{1}\cdots \delta_{p}}\left(T\right)\right]={\left(\frac{T}{\mu_{\delta_{1}\cdots \delta_{p}}}\right)}^{\beta_{\delta_{1}\cdots \delta_{p}}}, \end{array}$$

where $N_{\delta_{1}\ldots \delta_{p}}\left(T\right)$ is the hierarchical (at level *p*) subsystem-specific counting process, which denotes the number of failures before time *T*. It follows that $\beta_{\delta_{1}\ldots \delta_{p}}$ and $\alpha_{\delta_{1}\ldots \delta_{p}}$ are orthogonal parameters.

The orthogonal reparametrization of the PLP model enables us to obtain a likelihood function whose parameters $\beta_{\delta_{1}\ldots \delta_{p}}$ and $\alpha_{\delta_{1}\ldots \delta_{p}}$ are independent with desirable properties. In this case, based on the time truncation design, the hierarchical (at level *p*) subsystem-specific likelihood function for *n* ≥ 1 failures observed at times *t*_1_ < *t*_2_ < ⋯ < *t*_*n*_ < *T* is given by

$$\begin{array}{c} \begin{array}{cc} L(\beta_{\delta_{1}\cdots \delta_{p}},\alpha_{\delta_{1}\cdots \delta_{p}}|n,t) & =c\beta_{\delta_{1}\cdots \delta_{p}}^{n}e^{-n\beta_{\delta_{1}\cdots \delta_{p}}/{\hat{\beta }}_{\delta_{1}\cdots \delta_{p}}}\alpha_{\delta_{1}\cdots \delta_{p}}^{n}e^{-\alpha_{\delta_{1}\cdots \delta_{p}}}\\ & \propto \gamma (\beta_{\delta_{1}\cdots \delta_{p}}|n+1,n/{\hat{\beta }}_{\delta_{1}\cdots \delta_{p}})\gamma (\alpha_{\delta_{1}\cdots \delta_{p}}|n+1,1), \end{array} \end{array}$$

where ***t*** = (*t*_1_, *t*_2_, …, *t*_*n*_) denotes the vector of failure times, $c=\prod_{i=1}^{n}t_{i}^{-1}$ and $\gamma \left(x|a,b\right)=\frac{b^{a}}{\Gamma (a)}x^{a-1}e^{-bx}$, for *x*, *a*, *b* > 0, is the probability density function of a gamma distribution with shape parameter *a* and scale parameter *b*. Moreover, ${\hat{\beta }}_{\delta_{1}\ldots \delta_{p}}$ is the (biased) maximum likelihood estimator (MLE) of $\beta_{\delta_{1}\ldots \delta_{p}}$, which is given by

$$\begin{array}{c} {\hat{\beta }}_{\delta_{1}\cdots \delta_{p}}=\frac{n}{\sum_{i=1}^{n}\log(\frac{T}{t_{i}})}. \end{array}$$

It is worth noting that *n*, *t*_*i*_ and **t** should also carry *δ*_1_…*δ*_*p*_ as a subscript (i.e., $n_{\delta_{1}\ldots \delta_{p}}$, $t_{i;\;\delta_{1}\ldots \delta_{p}}$ and $t_{\delta_{1}\ldots \delta_{p}}$), but we omit it so as not to clutter the notation.

For a further discussion on the advantages of having orthogonal parameters, see Cox and Reid [28].

## 4 Bayesian inference

In this paper, we investigate the repairable system in the presence of hierarchical competing risks via objective Bayesian approach. A non-informative prior is used to depict lack of prior knowledge about the quantity of interest. There are different ways to obtain objective priors for the parameters of our model. Although the Jeffreys prior is the most commonly used, this prior may not be adequate in multivariate case [18]. Tibshirani [29] proposed an alternative method to derive a class of objective priors *π*(*θ*_1_, *θ*_2_), where *θ*_1_ is the parameter of interest and *θ*_2_ is the nuisance parameter, so that the credible interval for *θ*_1_ has a coverage error *O*(*n*^−1^) in the frequentist sense, i.e.,

$$\begin{array}{c} P[\theta_{1}\leq \theta_{1}^{1-\xi }(\pi ;t)|(\theta_{1},\theta_{2})]=1-\xi -O(n^{-1}), \end{array}$$ (2)

where $\theta_{1}^{1-\xi }(\pi ;t)|(\theta_{1},\theta_{2})$ denotes the (1 − *ξ*)-th quantile of the posterior distribution of *θ*_1_. The priors that satisfy (2) are known as matching priors.

Let $I_{\theta_{1},\theta_{2}}(\theta_{1},\theta_{2})$ denote the (*θ*_1_, *θ*_2_) entry of the Fisher information matrix *I*(*θ*_1_, *θ*_2_). To obtain such priors, Tibshirani [29] showed that if *θ*_1_ and *θ*_2_ are orthogonal parameters in the sense discussed by Cox and Reid [28], i.e., $I_{\theta_{1},\theta_{2}}(\theta_{1},\theta_{2})=0$, where *θ*_1_ is the parameter of interest and *θ*_2_ is the orthogonal nuisance parameter, then the matching priors are all priors of the form

$$\begin{array}{c} \pi (\theta_{1},\theta_{2})=g(\theta_{2})\sqrt{I_{\theta_{1},\theta_{1}}(\theta_{1},\theta_{2})}, \end{array}$$ (3)

where *g*(*θ*_2_) > 0 is an arbitrary function and $I_{\theta_{1},\theta_{1}}(\theta_{1},\theta_{2})$ is the *θ*_1_ entry of the Fisher information matrix. Tibshirani [29] showed that (3) is also a matching prior when *θ*_2_ is a vector of nuisance parameters.

Considering the proposed model, and assuming that *δ*_1_ = {1, …, *N*_1_}, …, *δ*_*p*_ = {1, …, *N*_*p*_(*δ*_1_, …, *δ*_*p*−1_)}, the elements of the Fisher information matrix can be expressed as

$$\begin{array}{cc} I_{\beta_{\delta_{1}\cdots \delta_{p}},\beta_{\delta_{1}\cdots \delta_{p}}}(\beta_{\delta_{1}\cdots \delta_{p}},\alpha_{\delta_{1}\cdots \delta_{p}}) & =\alpha_{\delta_{1}\cdots \delta_{p}}\beta_{\delta_{1}\cdots \delta_{p}}^{-2},\\ I_{\alpha_{\delta_{1}\cdots \delta_{p}},\alpha_{\delta_{1}\cdots \delta_{p}}}(\beta_{\delta_{1}\cdots \delta_{p}},\alpha_{\delta_{1}\cdots \delta_{p}}) & =\alpha_{\delta_{1}\cdots \delta_{p}}^{-1},\\ I_{\beta_{\delta_{1}\cdots \delta_{p}},\alpha_{\delta_{1}\cdots \delta_{p}}}(\beta_{\delta_{1}\cdots \delta_{p}},\alpha_{\delta_{1}\cdots \delta_{p}}) & =0,\\ I_{\alpha_{\delta_{1}\cdots \delta_{p}},\beta_{\delta_{1}\cdots \delta_{p}}}(\beta_{\delta_{1}\cdots \delta_{p}},\alpha_{\delta_{1}\cdots \delta_{p}}) & =0. \end{array}$$

From (3), one of the possible solutions is given by

$$\begin{array}{c} \pi (\beta ,\alpha )=\prod_{\delta_{1}=1}^{N_{1}}\cdots \prod_{\delta_{p}=1}^{N_{p}}\frac{1}{\beta_{\delta_{1}\cdots \delta_{p}}\sqrt{\alpha_{\delta_{1}\cdots \delta_{p}}}}, \end{array}$$ (4)

where $\beta =(\beta_{1\ldots 1},\ldots ,\beta_{N_{1}\ldots N_{p}})$ and $\alpha =(\alpha_{1\ldots 1},\ldots ,\alpha_{N_{1}\ldots N_{p}})$.

The prior given above satisfies (3) for all $\beta_{\delta_{1}\ldots \delta_{p}}$ and $\alpha_{\delta_{1}\ldots \delta_{p}}$ selected as interested parameters. Hence, the obtained prior is a matching prior for all the parameters, which implies that the credibility interval for any parameter has a coverage error *O*(*n*^−1^) in the frequentist sense.

Another important objective prior is the reference prior introduced by Bernardo [13] with further developments by Berger and Bernardo [16, 17]. This prior is defined as the prior that maximizes the expected Kullback-Leibler distance between the posterior distribution and the prior distribution based on the experimental data. Bernardo [18] proved that the reference prior has desirable properties, such as invariance, consistency under marginalization and consistent sampling properties. If the parameters of the model are orthogonal, the following lemma (see Berger *et al.* [30]) can be used to easily obtain a one-at-a-time reference prior to any chosen parameter of interest and any ordering of the nuisance parameters (hereafter referred to as overall reference prior).

**Lemma 4.1***Consider the unknown parameters****θ*** = (*θ*_1_, *θ*_2_) *with associated Fisher information matrix I*(*θ*_1_, *θ*_2_). *If* *I*(*θ*_1_, *θ*_2_) *is of the form*

$$\begin{array}{c} I(\theta_{1},\theta_{2})=\text{diag}(f(\theta_{1})g(\theta_{2}),h(\theta_{2})w(\theta_{1})), \end{array}$$

*where f*, *g*, *h and w are positive functions of* ***θ***, *then the overall reference prior is given by*

$$\begin{array}{c} \pi_{R}(\theta_{1},\theta_{2})=\sqrt{f(\theta_{1})h(\theta_{2})}\;. \end{array}$$ (5)

Assuming that $\theta \in R^{k}$, where *k* is the number of parameters, the same approach can be applied to obtain the overall reference prior related to the vector of parameters. Here, we have that $f_{\delta_{1}\ldots \delta_{p}}(\beta_{\delta_{1}\ldots \delta_{p}})=\beta_{\delta_{1}\ldots \delta_{p}}^{-2}$ and $h_{\delta_{1}\ldots \delta_{p}}(\beta_{\delta_{1}\ldots \delta_{p}})=\alpha_{\delta_{1}\ldots \delta_{p}}^{-1}$. Hence, from (5), the overall reference prior is given by

$$\begin{array}{c} \pi_{R}(\beta ,\alpha )=\prod_{\delta_{1}=1}^{N_{1}}\cdots \prod_{\delta_{p}=1}^{N_{p}}\frac{1}{\beta_{\delta_{1}\cdots \delta_{p}}\sqrt{\alpha_{\delta_{1}\cdots \delta_{p}}}}. \end{array}$$

Therefore, the prior (4) is an overall reference prior and also a matching prior for all the parameters. The obtained posterior distribution is given by

$$\begin{array}{c} \pi_{R}(\beta ,\alpha |n,t)\propto \\ \propto \prod_{\delta_{1}=1}^{N_{1}}\cdots \prod_{\delta_{p}=1}^{N_{p}}\gamma (\beta_{\delta_{1}\cdots \delta_{p}}|n_{\delta_{1}\cdots \delta_{p}},n_{\delta_{1}\cdots \delta_{p}}/{\hat{\beta }}_{\delta_{1}\cdots \delta_{p}})\gamma (\alpha_{\delta_{1}\cdots \delta_{p}}|n_{\delta_{1}\cdots \delta_{p}}+\frac{1}{2},1), \end{array}$$

where $n=(n_{1\ldots 1},\ldots ,n_{N_{1}\ldots N_{p}})$.

Due to the consistent marginalization property of the overall reference prior, the marginal reference posteriors are given by

$$\begin{array}{c} \pi_{R}\left(\beta_{\delta_{1}\cdots \delta_{p}}|n_{\delta_{1}\cdots \delta_{p}},t\right)\propto \gamma (\beta_{\delta_{1}\cdots \delta_{p}}|n_{\delta_{1}\cdots \delta_{p}},n_{\delta_{1}\cdots \delta_{p}}/{\hat{\beta }}_{\delta_{1}\cdots \delta_{p}}) \end{array}$$

and

$$\begin{array}{c} \pi_{R}\left(\alpha_{\delta_{1}\cdots \delta_{p}}|n_{\delta_{1}\cdots \delta_{p}},t\right)\propto \gamma (\alpha_{\delta_{1}\cdots \delta_{p}}|n_{\delta_{1}\cdots \delta_{p}}+\frac{1}{2},1). \end{array}$$

From the marginal posterior distribution, we can obtain the Bayes estimator assuming some rule, such as the posterior mean, median or mode. Here, we assume the posterior mode, also known as MAP estimator, since this approach leads to an unbiased estimator for $\beta_{\delta_{1}\ldots \delta_{p}}$ in the frequentist sense. The Bayes (MAP) estimator for $\beta_{\delta_{1}\ldots \delta_{p}}$ is given by

$$\begin{array}{c} {\hat{\beta }}_{\delta_{1}\cdots \delta_{p}}^{B}=\frac{n_{\delta_{1}\cdots \delta_{p}}-1}{\sum_{i=1}^{n}\log(\frac{T}{t_{i}})I_{\delta_{1}\cdots \delta_{p}}(t_{i})}, \end{array}$$

where $I_{\delta_{1}\ldots \delta_{p}}(t_{i})$ is the indicator function that equals one if the observation *t*_*i*_ belongs to the subsystem *δ*_1_⋯*δ*_*p*_, and *n* is the total number of failures that have occurred in the time interval (0, *T*] (as already stated in Section 3). From the estimator above, we have that $E[{\hat{\beta }}_{\delta_{1}\ldots \delta_{p}}^{B}]=\beta_{\delta_{1}\ldots \delta_{p}}$, i.e., such a Bayes estimator is unbiased in the frequentist sense. On the other hand, for $\alpha_{\delta_{1}\ldots \delta_{p}}$, the Bayes (MAP) estimator is given by

$$\begin{array}{c} {\hat{\alpha }}_{\delta_{1}\cdots \delta_{p}}^{B}=n_{\delta_{1}\cdots \delta_{p}}-\frac{1}{2}. \end{array}$$

In this case, we have that

$$\begin{array}{c} E[{\hat{\alpha }}_{\delta_{1}\cdots \delta_{p}}^{B}]=\alpha_{\delta_{1}\cdots \delta_{p}}-\frac{1}{2}. \end{array}$$

Hence, such a Bayes estimator for $\alpha_{\delta_{1}\ldots \delta_{p}}$ has a systematic bias of −0.5. Once we have identified the bias, we can remove it. In this case, we will not have that MAP estimator, but a bias-corrected MAP (BMAP) estimator. Hereafter, we will consider the BMAP estimator for the model parameters, which will be computed by

$$\begin{array}{c} {\hat{\beta }}_{\delta_{1}\cdots \delta_{p}}^{\text{BC}}=\frac{n_{\delta_{1}\cdots \delta_{p}}-1}{\sum_{i=1}^{n}\log(\frac{T}{t_{i}})I_{\delta_{1}\cdots \delta_{p}}(t_{i})} \end{array}$$

and

$$\begin{array}{c} {\hat{\alpha }}_{\delta_{1}\cdots \delta_{p}}^{\text{BC}}=n_{\delta_{1}\cdots \delta_{p}}. \end{array}$$

Now, since the marginal posterior distributions have closed-form expressions, we have that the *υ* = 100(1 − *ξ*)% credibility intervals for $\beta_{\delta_{1}\ldots \delta_{p}}$ and $\alpha_{\delta_{1}\ldots \delta_{p}}$ can be obtained directly from the quantile function of the gamma distribution, that is,

$$\begin{array}{c} \text{CI}\left(\beta_{{}^{\circ}};\upsilon \right)=[\gamma_{\text{Q}}(n_{{}^{\circ}},\frac{n_{{}^{\circ}}}{{\hat{\beta }}_{{}^{\circ}}};\;\frac{\xi }{2})\;;\;\gamma_{\text{Q}}(n_{{}^{\circ}},\frac{n_{{}^{\circ}}}{{\hat{\beta }}_{{}^{\circ}}};\;1-\frac{\xi }{2})] \end{array}$$

and

$$\begin{array}{c} \text{CI}\left(\alpha_{{}^{\circ}};\upsilon \right)=[\gamma_{\text{Q}}(n_{{}^{\circ}}+\frac{1}{2},1;\;\frac{\xi }{2})\;;\;\gamma_{\text{Q}}(n_{{}^{\circ}}+\frac{1}{2},1;\;1-\frac{\xi }{2})], \end{array}$$

where ° denotes the index *δ*_1_ ⋯ *δ*_*p*_ and *γ*_Q_(*a*, *b*; *υ*) is the quantile function of the gamma distribution with shape parameter *a* and scale parameter *b*, and 0 ≤ *υ* ≤ 1. This quantile function is available in most of the standard statistical softwares. For example, in R it can be computed by using the qgamma function. Therefore, the exact confidence intervals for the model parameters can be obtained without the use of intensive computation.

## 5 Simulation

In this section, we carry out a simulation study to investigate and compare the performance of the proposed Bayes estimators. To evaluate the estimators’ behavior, two metrics are used: the mean relative estimate (MRE) and the root mean squared error (RMSE), which are calculated, respectively, by

$$\begin{array}{c} \text{MRE}({\hat{\theta }}_{w})=\frac{1}{M}\sum_{m=1}^{M}\frac{{\hat{\theta }}_{w}^{\left(m\right)}}{\theta_{w}} \end{array}$$

and

$$\begin{array}{c} \text{RMSE}({\hat{\theta }}_{w})=\sqrt{\frac{1}{M}\sum_{m=1}^{M}{\left({\hat{\theta }}_{w}^{\left(m\right)}-\theta_{w}\right)}^{2}}, \end{array}$$

for *w* = 1, …, *κ*, where *M* = 100,000 is the number of Monte Carlo simulations and $\theta =\left(\theta_{1},\ldots ,\theta_{\kappa }\right)=(\beta_{1\ldots 1},\ldots ,\beta_{N_{1}\ldots N_{p}},\alpha_{1\ldots 1},\ldots ,\alpha_{N_{1}\ldots N_{p}})$ denotes the parameter vector. Besides, ${\hat{\theta }}_{w}^{\left(m\right)}$ represents the estimate of *θ*_*w*_ obtained from sample *m*, for *m* = 1, …, *M*.

Through this approach, it is expected that good estimators return MREs close to one and RMSEs close to zero. On the other hand, the 90% credibility intervals, which are obtained directly from the 5% and 95% quantiles of the gamma posterior distributions, are expected to have coverage probabilities (CPs) near the nominal value of 90%.

By considering the well-known results regarding NHPPs [10], and also from the assumption that the failure modes are independent, we can generate the failure times, for each Monte Carlo replication, according to the steps described in Algorithm 5. All numerical computations and simulations were done using the R programming language [31]. Due to space constraints, the results are reported only for six scenarios. However, similar findings are obtained for other parameter choices.

**Algorithm 1:** Generator of random numbers from the proposed model.

**Input:**

*p*, *T*

*N*_1_, *N*_2_(*δ*_1_), …, *N*_*p*_(*δ*_1_, …, *δ*_*p*−1_)

$\beta =(\beta_{1\ldots 1},\ldots ,\beta_{N_{1}\ldots N_{p}})$

$\alpha =(\alpha_{1\ldots 1},\ldots ,\alpha_{N_{1}\ldots N_{p}})$

**Output:**

$\left\{\left(t,\psi \right)\right\}=\left\{\left(t_{1\ldots 1},\psi_{1\ldots 1}\right),\ldots ,\left(t_{N_{1}\ldots N_{p}},\psi_{N_{1}\ldots N_{p}}\right)\right\}$

**Procedure:**

**for***δ*_1_ ≔ 1 **to** *N*_1_ **do**

 **for** *δ*_2_ ≔ 1 **to** *N*_2_(*δ*_1_) **do**

  ⋮

  **for** *δ*_*p*_ ≔ 1 **to** *N*_*p*_(*δ*_1_, …, *δ*_*p*−1_) **do**

  

$$\begin{array}{c} n_{\delta_{1},\ldots ,\delta_{p}}∼\mathrm{Poisson}\left(\alpha_{\delta_{1},\ldots ,\delta_{p}}\right) \end{array}$$

   **for** *i* ≔ 1 **to** $n_{\delta_{1},\ldots ,\delta_{p}}$ **do**

   

$$\begin{array}{cc} U_{i,\delta_{1},\ldots ,\delta_{p}} & ∼\mathrm{Uniform}(0,1)\\ t_{i,\delta_{1},\ldots ,\delta_{p}} & =T\;U_{i,\delta_{1},\ldots ,\delta_{p}}^{1/\beta_{\delta_{1},\ldots ,\delta_{p}}}\\ \psi_{i,\delta_{1},\ldots ,\delta_{p}} & =\delta_{1},\ldots ,\delta_{p} \end{array}$$

   **end**

  **end**

   ⋮

 **end**

**end**

**Fig 4:**
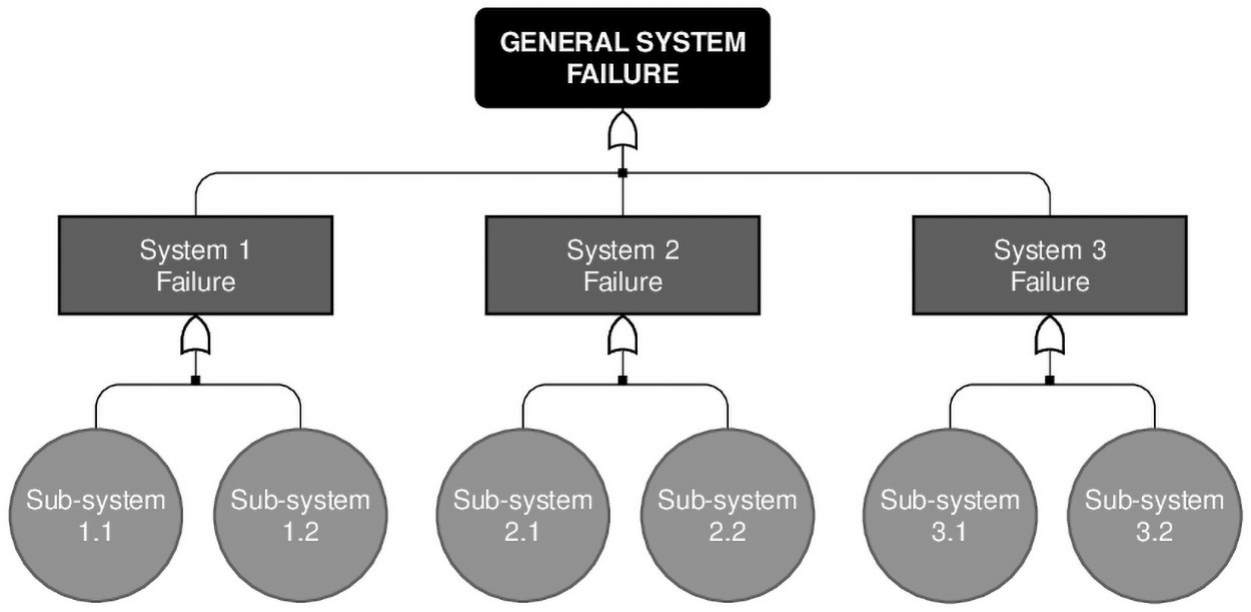
Proposed failure structure. Simulated failure structure for Scenarios 1, 2 and 3.

**Fig 5:**
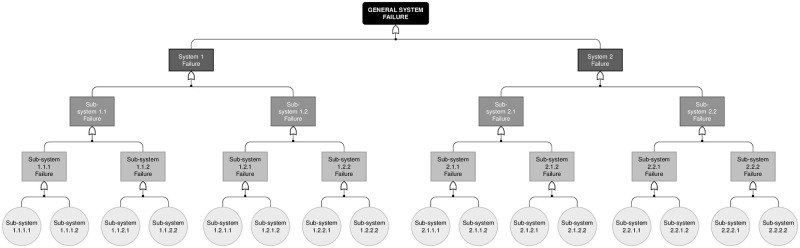
Proposed failure structure. Simulated failure structure for Scenarios 4, 5 and 6.

In what follows, we present the results for two distinct structures of a single system, both under the assumption that the components system is observed in the fixed time interval (0, *T*], where *T* = 60. The first is a system subject to 3 failure causes each with 2 subcauses; and the second is a system subject to 2 main causes each one subject to 2 subcauses which, in turn, are subject to 2 other causes which, in the end, are also subject to 2 causes, in a 4-level structure. These structures can be seen in Figs 4 and 5.

The parameters set for each underlying cause is presented in Table 1.

**Table 1:** Proposed scenarios for simulation.

**Scenario 1**			**Scenario 2**			**Scenario 3**		
**System**	***β***	***α***	**System**	***β***	***α***	**System**	***β***	***α***
1.1	1.5	22	1.1	2.2	30	1.1	0.20	35
1.2	2.3	18	1.2	0.7	25	1.2	0.50	24
2.1	1.2	20	2.1	1.5	27	2.1	0.90	18
2.2	3.5	25	2.2	0.2	32	2.2	1.00	20
3.1	1.0	21	3.1	1.0	23	3.1	0.80	32
3.2	1.1	23	3.2	1.8	20	3.2	0.05	25
**Scenario 4**			**Scenario 5**			**Scenario 6**		
**System**	***β***	***α***	**System**	***β***	***α***	**System**	***β***	***α***
1.1.1.1	1.3	20	1.1.1.1	1.0	20	1.1.1.1	0.10	20
1.1.1.2	1.5	22	1.1.1.2	1.2	25	1.1.1.2	0.10	22
1.1.2.1	2.0	23	1.1.2.1	0.8	26	1.1.2.1	0.50	30
1.1.2.2	1.8	25	1.1.2.2	1.5	23	1.1.2.2	0.30	28
1.2.1.1	1.1	22	1.2.1.1	0.3	18	1.2.1.1	0.90	18
1.2.1.2	2.5	21	1.2.1.2	1.8	17	1.2.1.2	0.80	25
1.2.2.1	1.2	30	1.2.2.1	0.7	23	1.2.2.1	0.05	19
1.2.2.2	1.5	32	1.2.2.2	1.5	22	1.2.2.2	0.40	17
2.1.1.1	1.0	17	2.1.1.1	0.5	20	2.1.1.1	0.20	20
2.1.1.2	1.1	18	2.1.1.2	0.9	17	2.1.1.2	0.01	26
2.1.2.1	1.0	20	2.1.2.1	2.0	28	2.1.2.1	0.30	32
2.1.2.2	2.1	18	2.1.2.2	1.0	22	2.1.2.2	0.70	19
2.2.1.1	1.6	29	2.2.1.1	0.4	19	2.2.1.1	0.40	23
2.2.1.2	1.5	25	2.2.1.2	0.1	23	2.2.1.2	0.90	25
2.2.2.1	2.5	24	2.2.2.1	1.4	20	2.2.2.1	0.10	26
2.2.2.2	3.0	23	2.2.2.2	1.9	18	2.2.2.2	0.20	20

Scenarios for a single system subject to 3 failure causes each one with 2 subcauses (Scenarios 1, 2 and 3); and a single system subject to 2 failure causes each one with 2 subcauses which, in turn, have 2 causes of failure and, finally, also 2 other causes of failure (Scenarios 4, 5 and 6).

As can be seen in Tables 2 and 3, the MREs are very close to one, with no exception, especially for the BMAP estimator. On the other hand, the observed values of RMSE are, in general, less than 0.5 for the MAP/BMAP estimator of ***β*** and less than 5 in the case of ***α***. The CPs are close to the nominal value of 90%, especially in the case of the exact intervals (CB) when compared to the asymptotic ones (B).

**Table 2:** Simulation results for scenarios 1, 2 and 3.

Parameter	Scenario 1							Scenario 2							Scenario 3						
	Value	MRE		RMSE		CP		Value	MRE		RMSE		CP		Value	MRE		RMSE		CP	
		B	CB	B	CB	B	CB		B	CB	B	CB	B	CB		B	CB	B	CB	B	CB
*α*_1.1_	22.0	0.977	1.000	4.719	4.693	0.856	0.892	30.0	0.983	1.000	5.505	5.483	0.887	0.899	35.00	0.986	1.000	5.942	5.921	0.871	0.892
*β*_1.1_	1.5	1.000	1.000	0.348	0.348	0.885	0.899	2.2	1.000	1.000	0.427	0.427	0.888	0.898	0.20	0.999	0.999	0.035	0.035	0.890	0.899
*α*_1.2_	18.0	0.972	1.000	4.276	4.247	0.880	0.900	25.0	0.980	1.000	5.027	5.002	0.886	0.889	24.00	0.979	1.000	4.927	4.902	0.892	0.897
*β*_1.2_	2.3	1.000	1.000	0.599	0.599	0.884	0.900	0.7	1.001	1.001	0.151	0.151	0.887	0.900	0.50	1.000	1.000	0.110	0.110	0.886	0.899
*α*_2.1_	20.0	0.975	1.000	4.504	4.476	0.873	0.908	27.0	0.981	1.000	5.227	5.203	0.872	0.899	18.00	0.972	1.000	4.276	4.247	0.880	0.900
*β*_2.1_	1.2	1.000	1.000	0.294	0.294	0.885	0.898	1.5	1.001	1.001	0.309	0.309	0.888	0.899	0.90	1.000	1.000	0.234	0.234	0.884	0.900
*α*_2.2_	25.0	0.980	1.000	5.027	5.002	0.886	0.889	32.0	0.984	1.000	5.683	5.661	0.886	0.908	20.00	0.975	1.000	4.504	4.476	0.873	0.908
*β*_2.2_	3.5	1.001	1.001	0.753	0.753	0.887	0.900	0.2	1.000	1.000	0.037	0.037	0.889	0.898	1.00	1.000	1.000	0.245	0.245	0.885	0.898
*α*_3.1_	21.0	0.976	1.000	4.616	4.589	0.864	0.900	23.0	0.978	1.000	4.824	4.798	0.889	0.904	32.00	0.984	1.000	5.683	5.661	0.886	0.908
*β*_3.1_	1.0	1.000	1.000	0.238	0.238	0.885	0.898	1.0	1.000	1.000	0.226	0.226	0.886	0.899	0.80	1.000	1.000	0.150	0.150	0.889	0.898
*α*_3.2_	23.0	0.978	1.000	4.824	4.798	0.889	0.904	20.0	0.975	1.000	4.504	4.476	0.873	0.908	25.00	0.980	1.000	5.027	5.002	0.886	0.889
*β*_3.2_	1.1	1.000	1.000	0.248	0.248	0.886	0.899	1.8	1.000	1.000	0.440	0.440	0.885	0.898	0.05	1.001	1.001	0.011	0.011	0.887	0.900

MREs, RMSEs and CPs from the MAP (B) and BMAP (CB) estimators, considering different parameter values (Scenarios 1, 2 and 3).

**Table 3:** Simulation results for scenarios 4, 5 and 6.

Parameter	Scenario 4							Scenario 5							Scenario 6						
	Value	MRE		RMSE		CP		Value	MRE		RMSE		CP		Value	MRE		RMSE		CP	
		B	CB	B	CB	B	CB		B	CB	B	CB	B	CB		B	CB	B	CB	B	CB
*α*_1.1.1.1_	20.0	0.975	1.000	4.504	4.476	0.873	0.908	20.0	0.975	1.000	4.504	4.476	0.873	0.908	20.00	0.975	1.000	4.504	4.476	0.873	0.908
*β*_1.1.1.1_	1.3	1.000	1.000	0.318	0.318	0.885	0.898	1.0	1.000	1.000	0.245	0.245	0.885	0.898	0.10	1.000	1.000	0.024	0.024	0.885	0.898
*α*_1.1.1.2_	22.0	0.977	1.000	4.719	4.693	0.856	0.892	25.0	0.980	1.000	5.027	5.002	0.886	0.889	22.00	0.977	1.000	4.719	4.693	0.856	0.892
*β*_1.1.1.2_	1.5	1.000	1.000	0.348	0.348	0.885	0.899	1.2	1.001	1.001	0.258	0.258	0.887	0.900	0.10	1.000	1.000	0.023	0.023	0.885	0.899
*α*_1.1.2.1_	23.0	0.978	1.000	4.824	4.798	0.889	0.904	26.0	0.981	1.000	5.124	5.100	0.879	0.906	30.00	0.983	1.000	5.505	5.483	0.887	0.899
*β*_1.1.2.1_	2.0	1.000	1.000	0.451	0.451	0.886	0.899	0.8	1.001	1.001	0.169	0.169	0.887	0.899	0.50	1.000	1.000	0.097	0.097	0.888	0.898
*α*_1.1.2.2_	25.0	0.980	1.000	5.027	5.002	0.886	0.889	23.0	0.978	1.000	4.824	4.798	0.889	0.904	28.00	0.982	1.000	5.321	5.297	0.865	0.893
*β*_1.1.2.2_	1.8	1.001	1.001	0.387	0.387	0.887	0.900	1.5	1.000	1.000	0.338	0.338	0.886	0.899	0.30	1.000	1.000	0.061	0.061	0.888	0.899
*α*_1.2.1.1_	22.0	0.977	1.000	4.719	4.693	0.856	0.892	18.0	0.972	1.000	4.276	4.247	0.880	0.900	18.00	0.972	1.000	4.276	4.247	0.880	0.900
*β*_1.2.1.1_	1.1	1.000	1.000	0.255	0.255	0.885	0.899	0.3	1.000	1.000	0.078	0.078	0.884	0.900	0.90	1.000	1.000	0.234	0.234	0.884	0.900
*α*_1.2.1.2_	21.0	0.976	1.000	4.616	4.589	0.864	0.900	17.0	0.971	1.000	4.155	4.125	0.890	0.910	25.00	0.980	1.000	5.027	5.002	0.886	0.889
*β*_1.2.1.2_	2.5	1.000	1.000	0.596	0.596	0.885	0.898	1.8	1.000	1.000	0.486	0.486	0.882	0.899	0.80	1.001	1.001	0.172	0.172	0.887	0.900
*α*_1.2.2.1_	30.0	0.983	1.000	5.505	5.483	0.887	0.899	23.0	0.978	1.000	4.824	4.798	0.889	0.904	19.00	0.974	1.000	4.395	4.367	0.882	0.890
*β*_1.2.2.1_	1.2	1.000	1.000	0.233	0.233	0.888	0.898	0.7	1.000	1.000	0.158	0.158	0.886	0.899	0.05	1.000	1.000	0.013	0.013	0.884	0.899
*α*_1.2.2.2_	32.0	0.984	1.000	5.683	5.661	0.886	0.908	22.0	0.977	1.000	4.719	4.693	0.856	0.892	17.00	0.971	1.000	4.155	4.125	0.890	0.910
*β*_1.2.2.2_	1.5	1.000	1.000	0.281	0.281	0.889	0.898	1.5	1.000	1.000	0.348	0.348	0.885	0.899	0.40	1.000	1.000	0.108	0.108	0.882	0.899
*α*_2.1.1.1_	17.0	0.971	1.000	4.155	4.125	0.890	0.910	20.0	0.975	1.000	4.504	4.476	0.873	0.908	20.00	0.975	1.000	4.504	4.476	0.873	0.908
*β*_2.1.1.1_	1.0	1.000	1.000	0.270	0.270	0.882	0.899	0.5	1.000	1.000	0.122	0.122	0.885	0.898	0.20	1.000	1.000	0.049	0.049	0.885	0.898
*α*_2.1.1.2_	18.0	0.972	1.000	4.276	4.247	0.880	0.900	17.0	0.971	1.000	4.155	4.125	0.890	0.910	26.00	0.981	1.000	5.124	5.100	0.879	0.906
*β*_2.1.1.2_	1.1	1.000	1.000	0.286	0.286	0.884	0.900	0.9	1.000	1.000	0.243	0.243	0.882	0.899	0.01	0.989	0.989	0.002	0.002	0.880	0.903
*α*_2.1.2.1_	20.0	0.975	1.000	4.504	4.476	0.873	0.908	28.0	0.982	1.000	5.321	5.297	0.865	0.893	32.00	0.984	1.000	5.683	5.661	0.886	0.908
*β*_2.1.2.1_	1.0	1.000	1.000	0.245	0.245	0.885	0.898	2.0	1.000	1.000	0.404	0.404	0.888	0.899	0.30	1.000	1.000	0.056	0.056	0.889	0.898
*α*_2.1.2.2_	18.0	0.972	1.000	4.276	4.247	0.880	0.900	22.0	0.977	1.000	4.719	4.693	0.856	0.892	19.00	0.974	1.000	4.395	4.367	0.882	0.890
*β*_2.1.2.2_	2.1	1.000	1.000	0.547	0.547	0.884	0.900	1.0	1.000	1.000	0.232	0.232	0.885	0.899	0.70	1.000	1.000	0.177	0.177	0.884	0.899
*α*_2.2.1.1_	29.0	0.983	1.000	5.413	5.390	0.893	0.905	19.0	0.974	1.000	4.395	4.367	0.882	0.890	23.00	0.978	1.000	4.824	4.798	0.889	0.904
*β*_2.2.1.1_	1.6	1.000	1.000	0.316	0.316	0.888	0.899	0.4	1.000	1.000	0.101	0.101	0.884	0.899	0.40	1.000	1.000	0.090	0.090	0.886	0.899
*α*_2.2.1.2_	25.0	0.980	1.000	5.027	5.002	0.886	0.889	23.0	0.978	1.000	4.824	4.798	0.889	0.904	25.00	0.980	1.000	5.027	5.002	0.886	0.889
*β*_2.2.1.2_	1.5	1.001	1.001	0.323	0.323	0.887	0.900	0.1	1.000	1.000	0.023	0.023	0.886	0.899	0.90	1.001	1.001	0.194	0.194	0.887	0.900
*α*_2.2.2.1_	24.0	0.979	1.000	4.927	4.902	0.892	0.897	20.0	0.975	1.000	4.504	4.476	0.873	0.908	26.00	0.981	1.000	5.124	5.100	0.879	0.906
*β*_2.2.2.1_	2.5	1.000	1.000	0.551	0.551	0.886	0.899	1.4	1.000	1.000	0.343	0.343	0.885	0.898	0.10	1.001	1.001	0.021	0.021	0.887	0.899
*α*_2.2.2.2_	23.0	0.978	1.000	4.824	4.798	0.889	0.904	18.0	0.972	1.000	4.276	4.247	0.880	0.900	20.00	0.975	1.000	4.504	4.476	0.873	0.908
*β*_2.2.2.2_	3.0	1.000	1.000	0.677	0.677	0.886	0.899	1.9	1.000	1.000	0.494	0.494	0.884	0.900	0.20	1.000	1.000	0.049	0.049	0.885	0.898

MREs, RMSEs and CPs from the MAP (B) and BMAP (CB) estimators, considering different parameter values (Scenarios 4, 5 and 6).

## 6 Applications

In Louzada *et al.* [25], the Annelida’s traction subsystem was used to illustrate the methodology discussed at the time. With the advance in the development of the robotic unit, the applications exposed here refine the previous one with the inclusion of more details about this important subsystem (Section 6.2), and also adding the new results obtained in this paper to another subsystem as well (pressure vessel subsystem in Section 6.1). The information available so far comes from their respective FMEA tables, which were reviewed by the technical team to further deepen the knowledge they have about their idealizations and tests already carried out.

In both subsystems, the parameters of the model proposed here were associated with the Severity (S), Occurrence (O) and Detectability (D) indices. Thus, it was possible to take random realizations (based on Algorithm 1) of the failure times that represent the perspective provided by the technical team at FMEA, from the perspective of reliability. This approach ensures the addition of information at this early stage of the project. It was also assumed that both subsystems are observed in the fixed time interval (0, *T*], where *T* = 209 days, which relates to an operating time close to 5,000 hours.

The current reliability requirements for both subsystems include, among numerous other factors, that: (i) with high probability, the system must remain in operation (without any failure) for at least a minimum number of days; (ii) on the other hand, the median lifetime of the first failure is expected to be around another number of days. These requirements were determined by considering the expected number of annual missions, the size of the step taken by the robotic unit inside the oil pipelines, its respective speed and the estimated time for the hydrate block to melt. Such estimates also allowed to assess the time needed per mission and, therefore, the minimum desired lifetime.

### 6.1 In-pipe robot—Pressure vessel set

**Fig 6:**
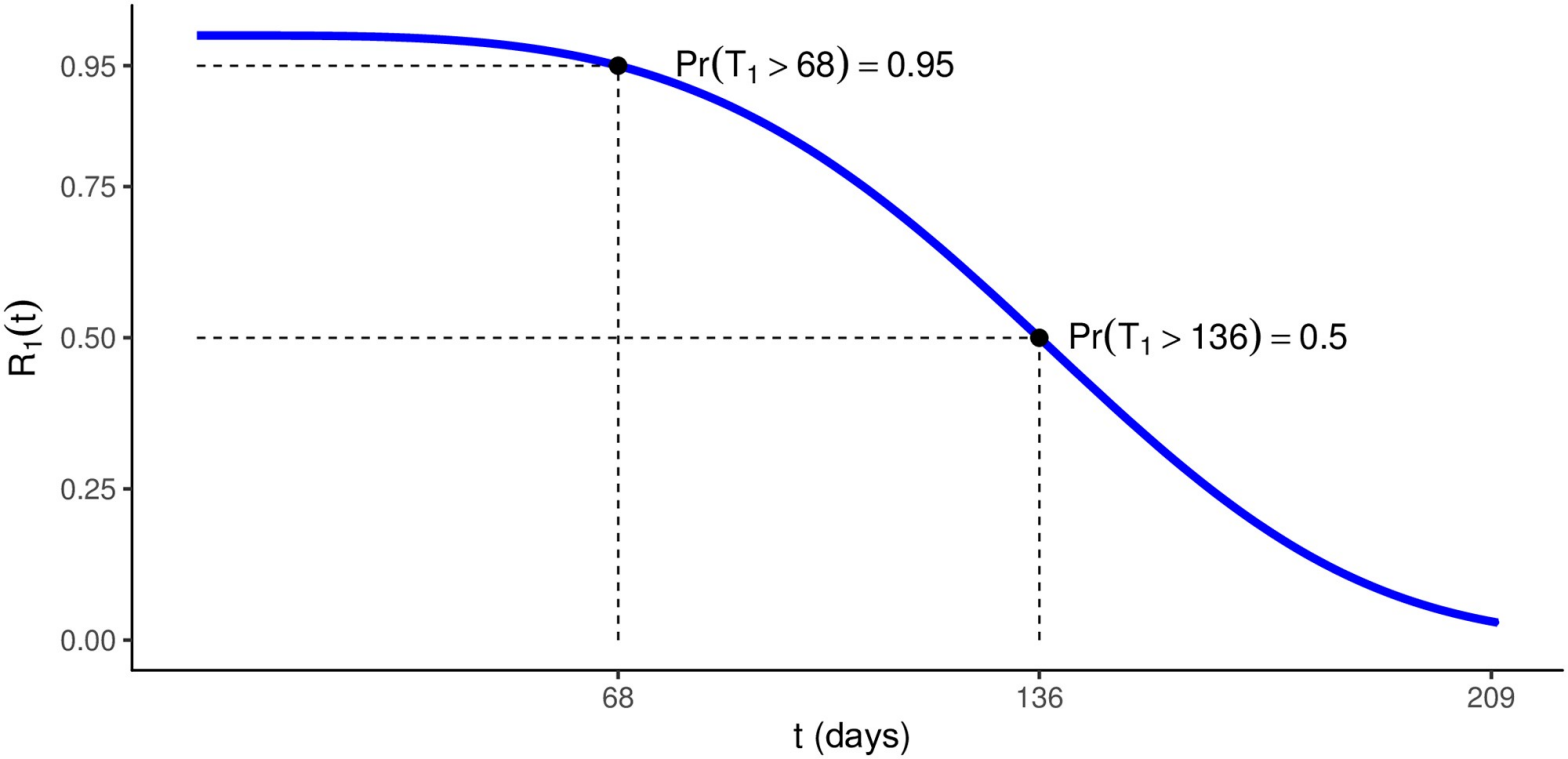
Reliability requirements. Pressure vessel set’s.

Based on the reliability requirements for the pressure vessel system, we consider that with high probability (≈95%) the first failure should not occur before 68 days. Similarly, the median of first-failure time should be around 136 days. In this way, we can plot the reliability curve associated with the proposed model (Fig 6). This curve is a partial reference for the reliability that we want to achieve at the end of the development of this system, in the light of the currently proposed modeling. We will see that all the uncertainty involved in this initial stage of the project exposes us to how far we are from this objective. However, it does allow for more targeted research routes.

The project designed 11 pressure vessels connected by connecting cables, as shown in Fig 2. Although the whole set of vessels will be exposed to the same environmental characteristics, their functions have different purposes and importances. However, the individual evaluation of the vessels will not be considered in this work, so that the same FMEA will represent, here, all 11 subsystems and the indices used, as well as the FTA, are shown in Table 4.

**Table 4:** FTA (with FMEA indices) for the in-pipe robot’s pressure vessel system.

				S	O	D
All Pressure Vessel	Pressure Vessel Set $\left({}_{these\;subsystems}^{there\;are\;11\;of}\right)$	Cable Conductor		7	7	5
		Pressure Vessel	Rear Vessel Cap	8	5	4
			Heatpipe	6	5	3
			Carbon Fiber Protection	8	4	7
			Vessel Base	8	4	7
			Front Vessel Cap	7	4	4

S = Severity, O = Occurrence, D = Detection.

An illustration of the data set generated can be obtained in full via an individual request for the authors. Due to the approximately linear behavior in the Duane plots, for each failure mode (see S1 Fig), we have indications that the theoretical PLP model may be appropriate to model a problem like this.

**Fig 7:**
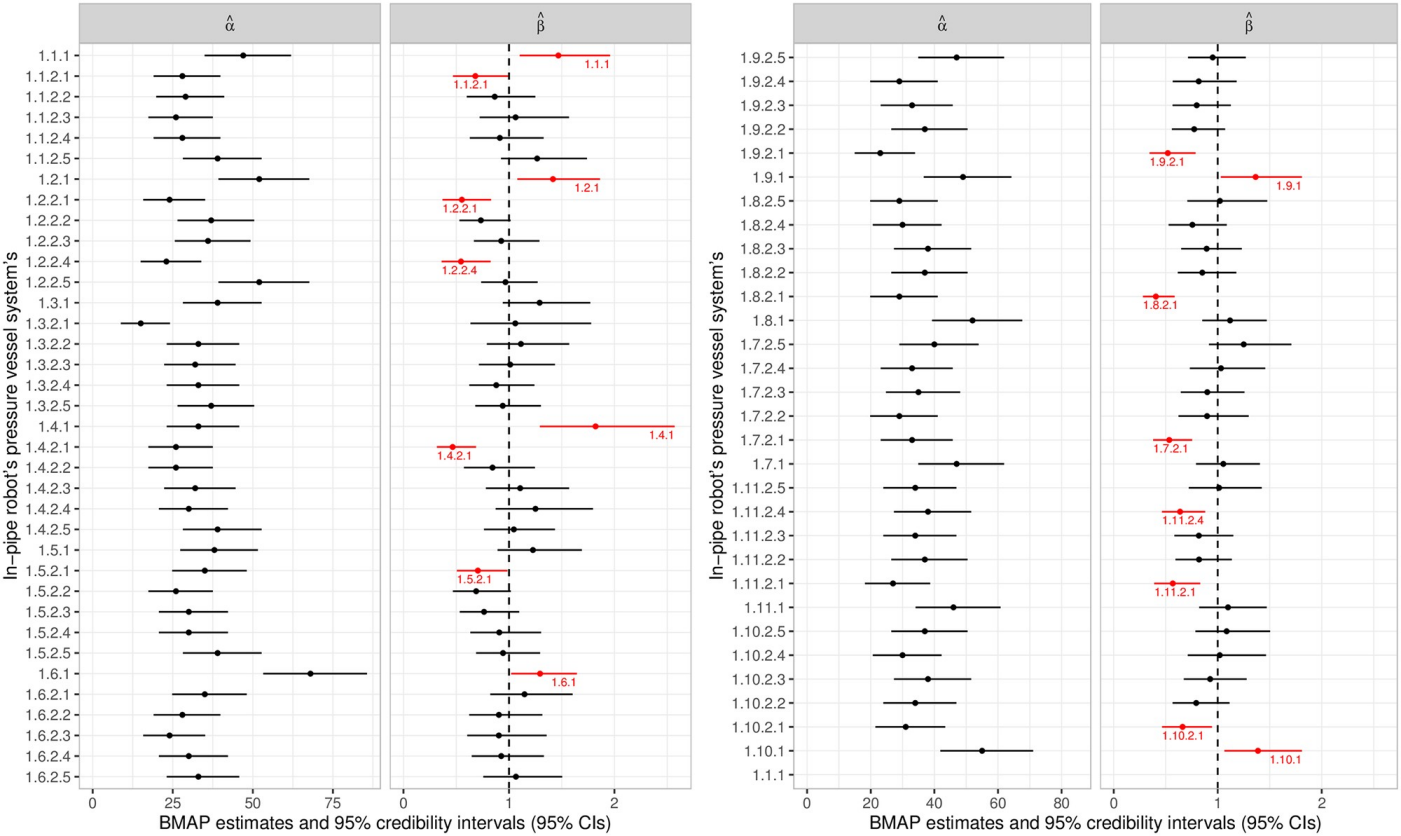
Parameters estimatives for the in-pipe robot’s pressure vessel system’s failure data. BMAP estimates and 95% credibility intervals (95% CIs) for the model parameters.

The summary for the parameter estimates (see Fig 7) express that, in a practical situation, the Cable Conductor subsystem would be undergoing a degradation process; there is statistical evidence of this in some cases (that is, in 6 out of 11 subsystems), and in others only indications (that is, in 5 out of 11 subsystems). On the other hand, the same occurs less frequently in the other subsystems.

**Fig 8:**
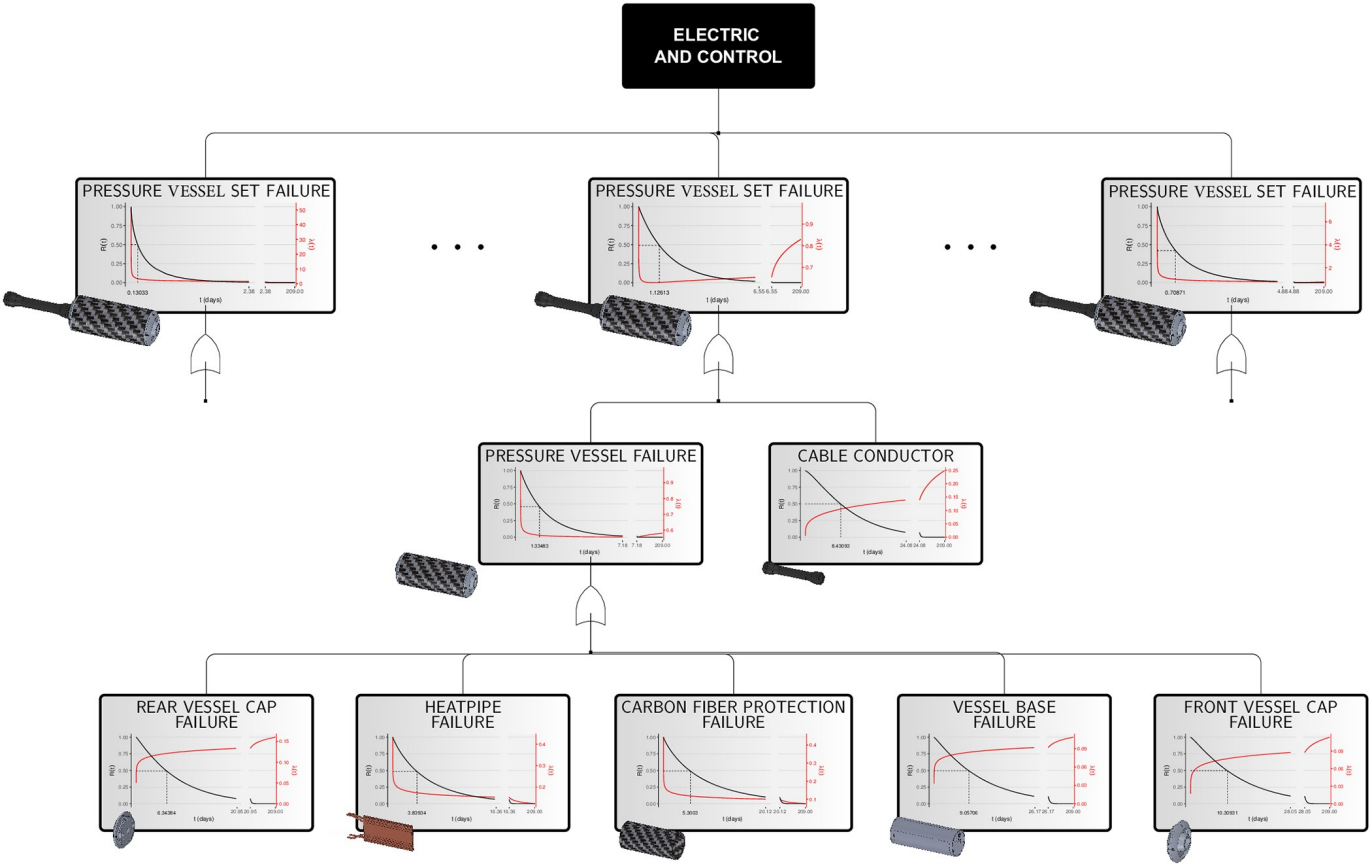
Estimated functions for the in-pipe robotic unit’s pressure vessel set system. First-failure time reliability (in black) and intensity (in red) functions by components, subsystems and systems.

Some subsystems did not have an increasing intensity function (which would indicate a degradation process), however, the failure intensity in the initial times was higher, which results in the high occurrence of failures in the first moments of activity and, therefore, significantly reduces the component’s survival time. This occurred more frequently in the Heatpipe and Carbon Fiber Protection subsystems. This information cannot be perceived directly on the parameter estimates (see Fig 7), however, the graphs associated with the first-failure time reliability and intensity curves, obtained by the adjustment, are shown in Fig 8.

From Fig 8, it is possible to notice that the desired reliability requirements are still far away. The median first-failure time of a pressure vessel is close to one day (depending on the randomization of the simulated data), which is still far from the desired 68 days. The result obtained by the model does not reflect any time observed in practice, however, it describes, from the perspective of the reliability analysis, all the uncertainty that still surrounds the development of this system component.

The graph of the observed number of failures *versus* the number of failures estimated over time can be used to assess the quality of the model’s fit. In S2 Fig, the plots for each component are presented and, in general, in a practical situation we would understand that the model was able to describe the observed behavior.

### 6.2 In-pipe robot—Traction system

In this section, we return to the situation described in Section 1. We obtained a suitable data set for this problem using a similar approach as proposed in the previous section, i.e., based on the limited but available information provided by the revised FMEA and FTA tools (see S3 Fig).

**Fig 9:**
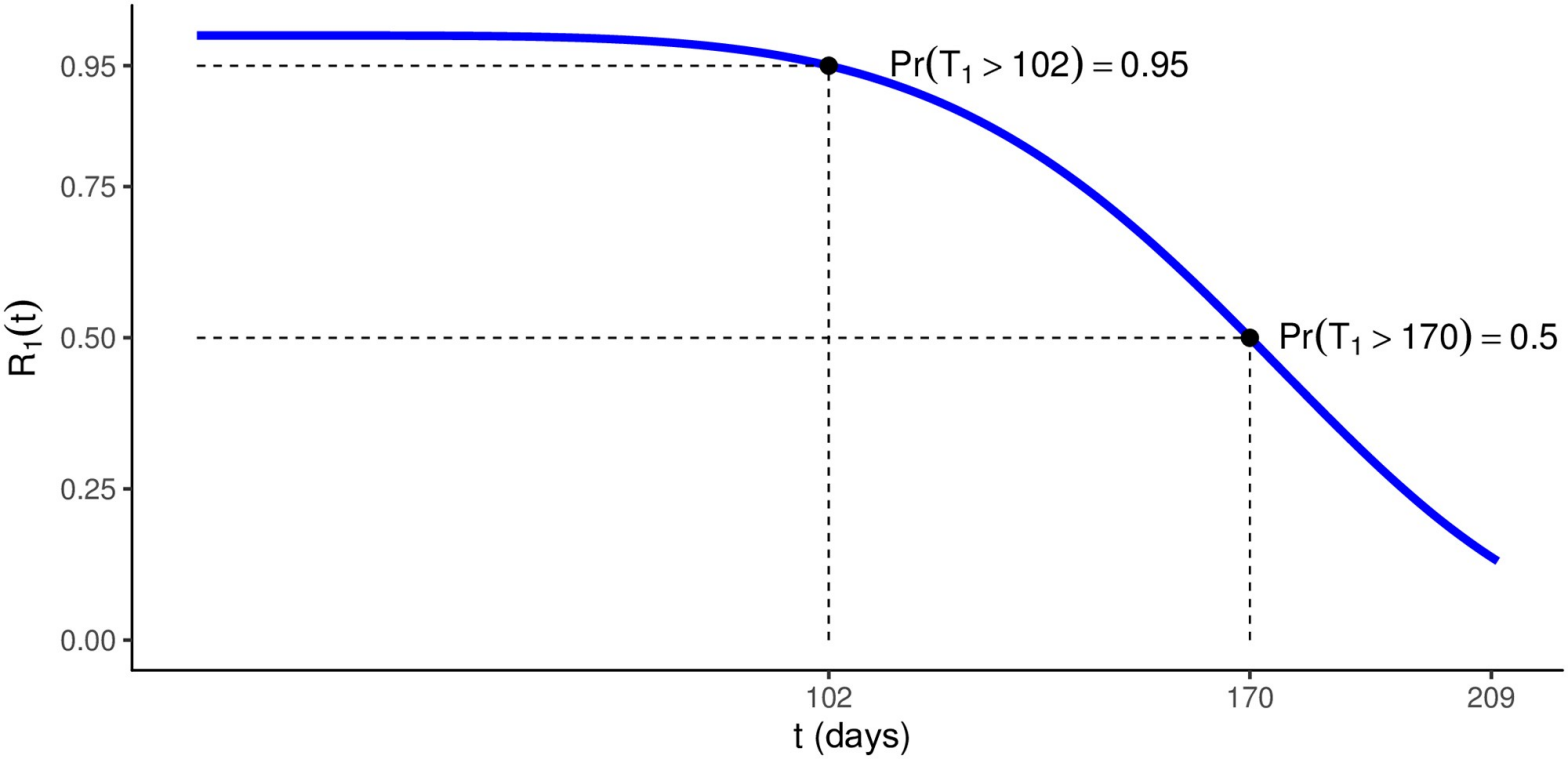
Reliability requirements. Traction system’s.

The required reliability for the traction system is graphically exposed in Fig 9, and claims, with high probability, that the system remains in operation for at least 102 days, without any failure. Also, the median failure time of the system has to be approximately 170 days.

The Duane plots built for each failure mode (see S4 Fig) have an approximately linear behavior, which demonstrates the theoretical suitability that a PLP model needs for adjustment.

**Fig 10:**
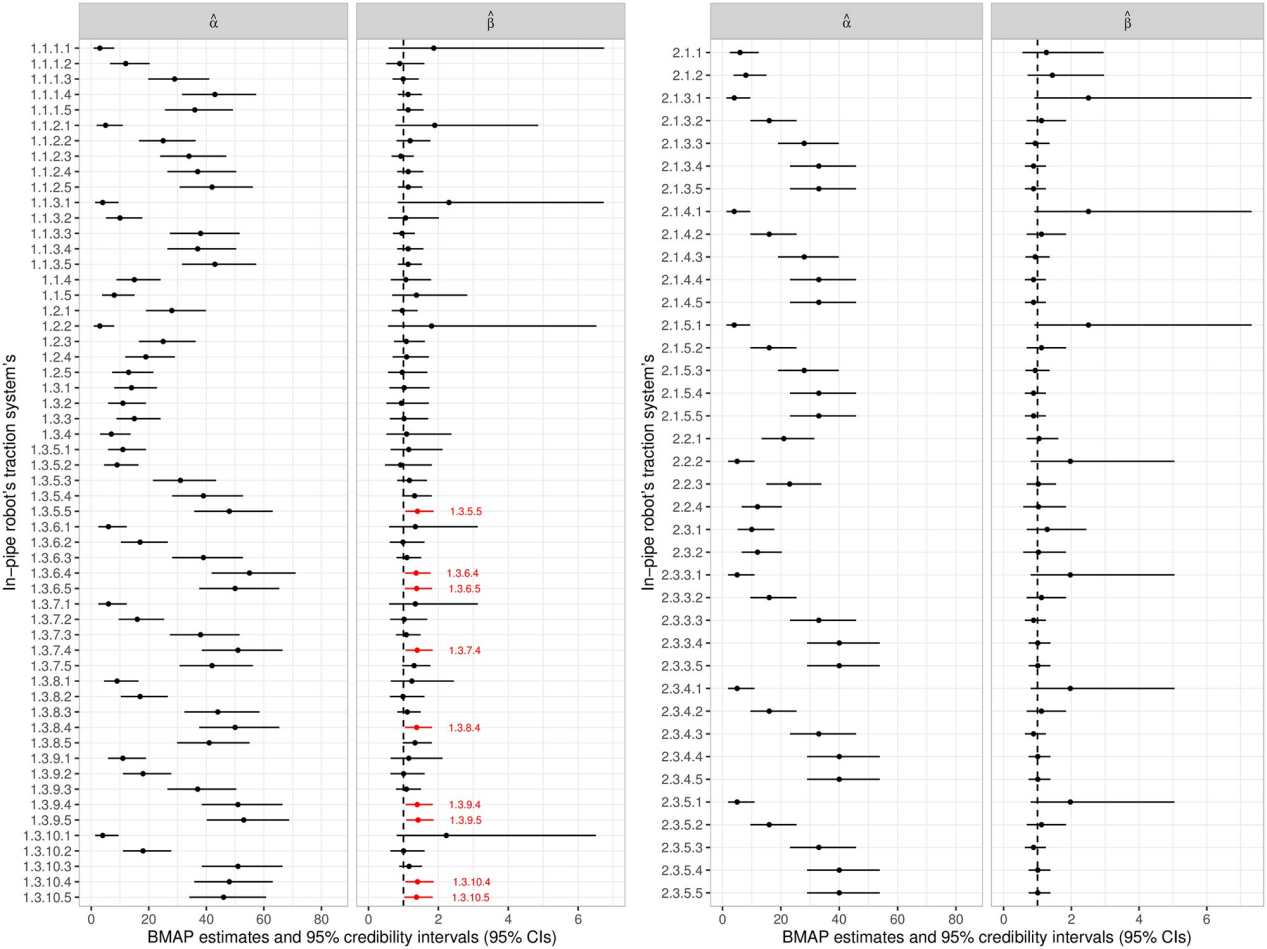
Parameters estimatives for the in-pipe robot’s traction system’s failure data. BMAP estimates and 95% credibility intervals (95% CIs) for the model parameters.

In a practical context, if the failure times came from some missions, the BMAP estimates of the adjusted model for the traction system (see Fig 10) would show that there are evidences of some components having an increasing failure intensity function (systems 1.3.5.5, 1.3.6.4–5, 1.3.7.4, 1.3.8.4, 1.3.9.4–5, 1.3.10.4–5); for many others, there are some indications; and for some, evidences of non-deterioration (systems 1.1.1.2–3, 1.1.2.3, 1.1.3.3, 1.2.1, 1.2.5, 1.3.2, 1.3.5.2, 1.3.6.2, 1.3.8.2, 2.1.3.3–5, 2.1.4.3–5, 2.1.5.3–5, 2.3.3.3, 2.3.4.3, 2.3.5.3).

From these results (see Fig 10), we identify that the components responsible for the main failure causes, which represent the biggest obstacles to reaching the reliability requirements, are the rubber (1.3.5–10.4) and adhesive (1.3.5–10.5) components. In particular, these units express an evident degradation behavior, which suggests the need for a preventive maintenance regime dedicated especially to these components, or even the renewal of the design idealized for the process performed by them. Indeed, the latter is what is currently being carried out, since the preliminary practical tests of the traction system exposed serious failures associated with the strength of the adhesive and the rubber to withstand the necessary force for the locomotion.

**Fig 11:**
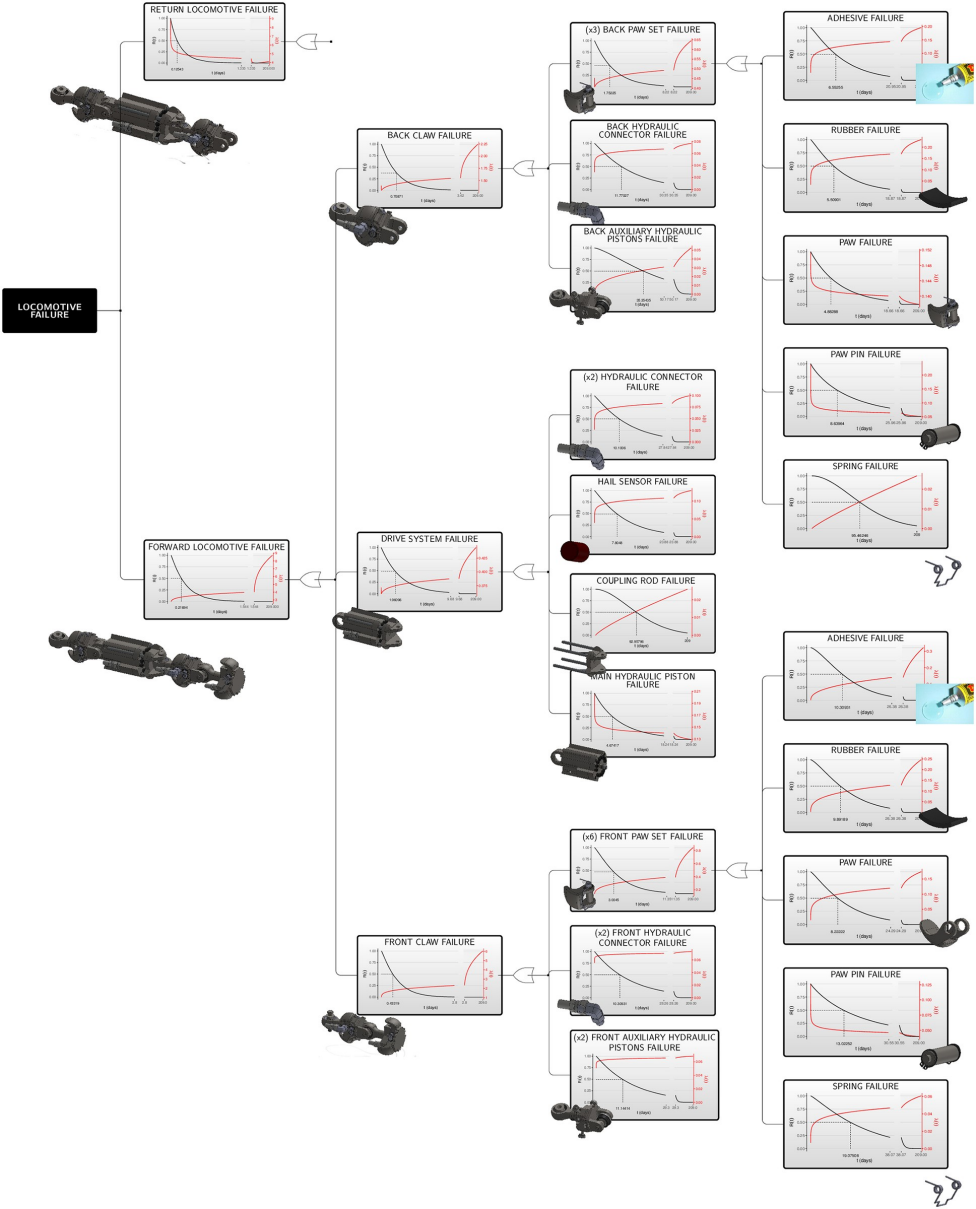
Estimated functions for the in-pipe robotic unit’s traction system. First-failure time reliability (in black) and intensity (in red) functions by components, subsystems and systems.

The estimated reliability and intensity functions for the first-failure times are shown in Fig 11. From this figure, it can be seen that the median first-failure time for some components, such as Spring (1.1.1–3.1) and Copling Rod (1.2.2), is high, with approximately 90 days. Other components, such as rubber (1.1.1–3.4), adhesive (1.1.1–3.5), paw (1.1.1–3.3) and main hydraulic piston (1.2.1), have a small first-failure median time, around 5 days. However, at the end of the interaction between all components and subsystems, the median time of the first-failure is around 0.12543 days for the return locomotive, and 0.21694 days for the forward locomotive.

The goodness of fit can be seen by the comparison between the observed and estimated number of failures along the time. These graphs, for each component, can be found in S5 Fig.

## 7 Concluding remarks and further research

In this paper, we have continued the study started in [25], presenting the model under consideration of an arbitrary number of hierarchical levels and maintaining the assumptions of competing risks with independent failure modes, in a minimal repair regime with a reparametrized PLP intensity function. In this context, we have obtained Bayesian estimators with corrected biases, as well as we have derived exact credibility intervals for the parameters. The properties of these estimators were evaluated in a simulation study, which returned good results.

The model structured in the proposed way allowed to highlight analytically and graphically the reliability associated with the first-failure times of each one of the subsystems (at any hierarchical level) of two arbitrary systems that illustrate the use of modeling. Namely, the pressure vessels set and the traction system of the developing system that has served as a practical motivation for this theoretical development.

As future works, we intend to evaluate the quality of these estimators in a context with outliers, their behavior (in terms of quality loss) when exposed to data from an imperfect repair regime. In addition, we wish to evaluate the change in reliability based on the increase in redundancy of some subsystems. We also intend to assume that repairs are either perfect or imperfect, and model the dependence among the failure modes via shared frailty models.

## Supporting information

S1 Fig Duane plots for the in-pipe robot’s pressure vessel system. For the failure modes.
(TIF)

S2 Fig Goodness of fit for the in-pipe robot’s pressure vessel system. Number of observed and estimated failures per component.
(TIF)

S3 Fig FTA (with FMEA indices) for the in-pipe robot’s traction system. S = Severity, O = Occurrence, D = Detection.
(TIF)

S4 Fig Duane plots for the in-pipe robot’s traction system. For the failure modes.
(TIF)

S5 Fig Goodness of fit for the in-pipe robot’s traction system. Number of observed and estimated failures per component.
(TIF)

S1 Data (ZIP)

S1 Raw images (PDF)
