# Supplementary figures and images for: Improved objective Bayesian estimator for a PLP model hierarchically represented subject to competing risks under minimal repair regime
Source: PLoS One. 2021 Aug 12;16(8):e0255944. doi: 10.1371/journal.pone.0255944 (PMC8360570; doi:10.1371/journal.pone.0255944)

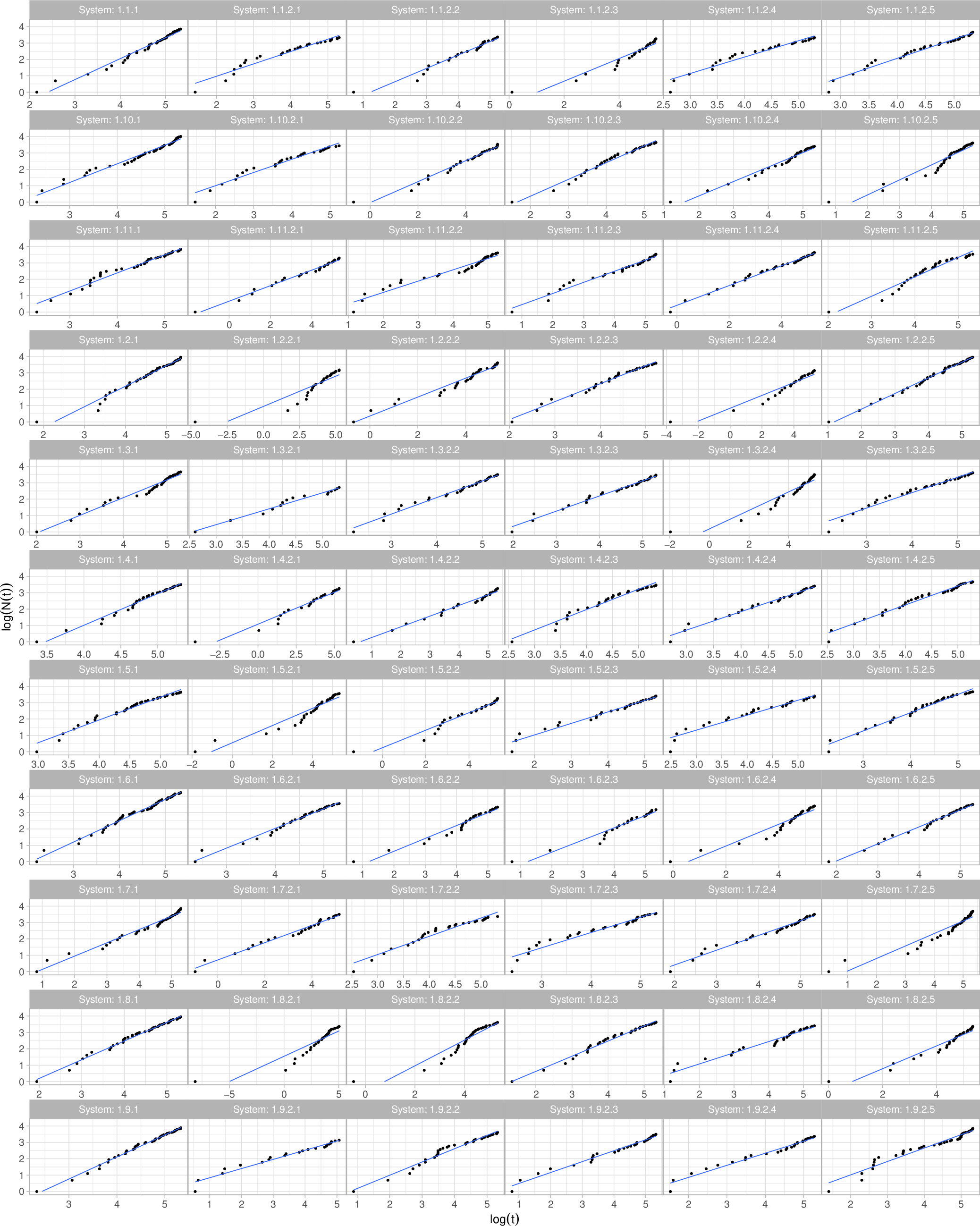

Supplement: S1 Fig — For the failure modes. (TIF) [file pone.0255944.s001.tif]

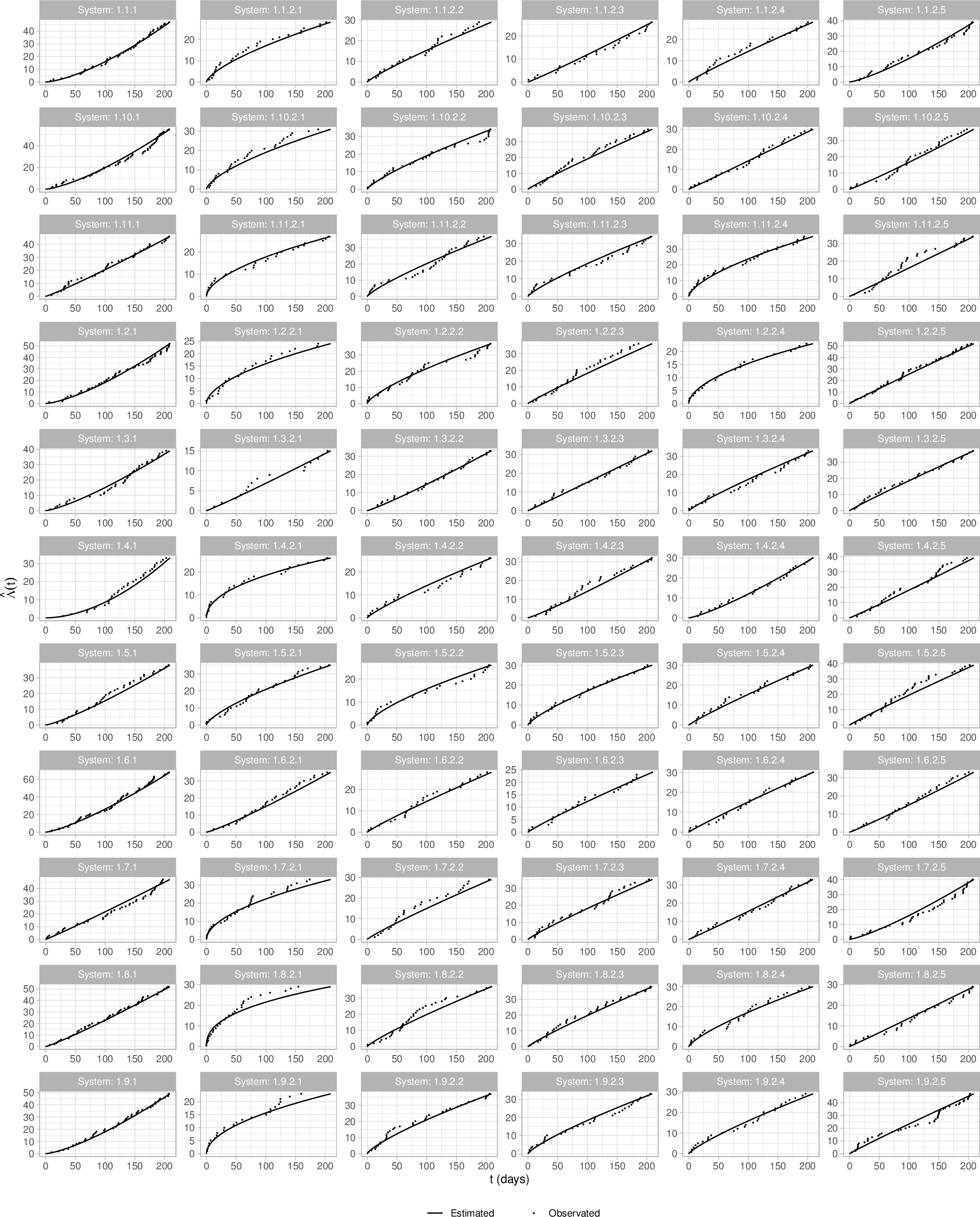

Supplement: S2 Fig — Number of observed and estimated failures per component. (TIF) [file pone.0255944.s002.tif]

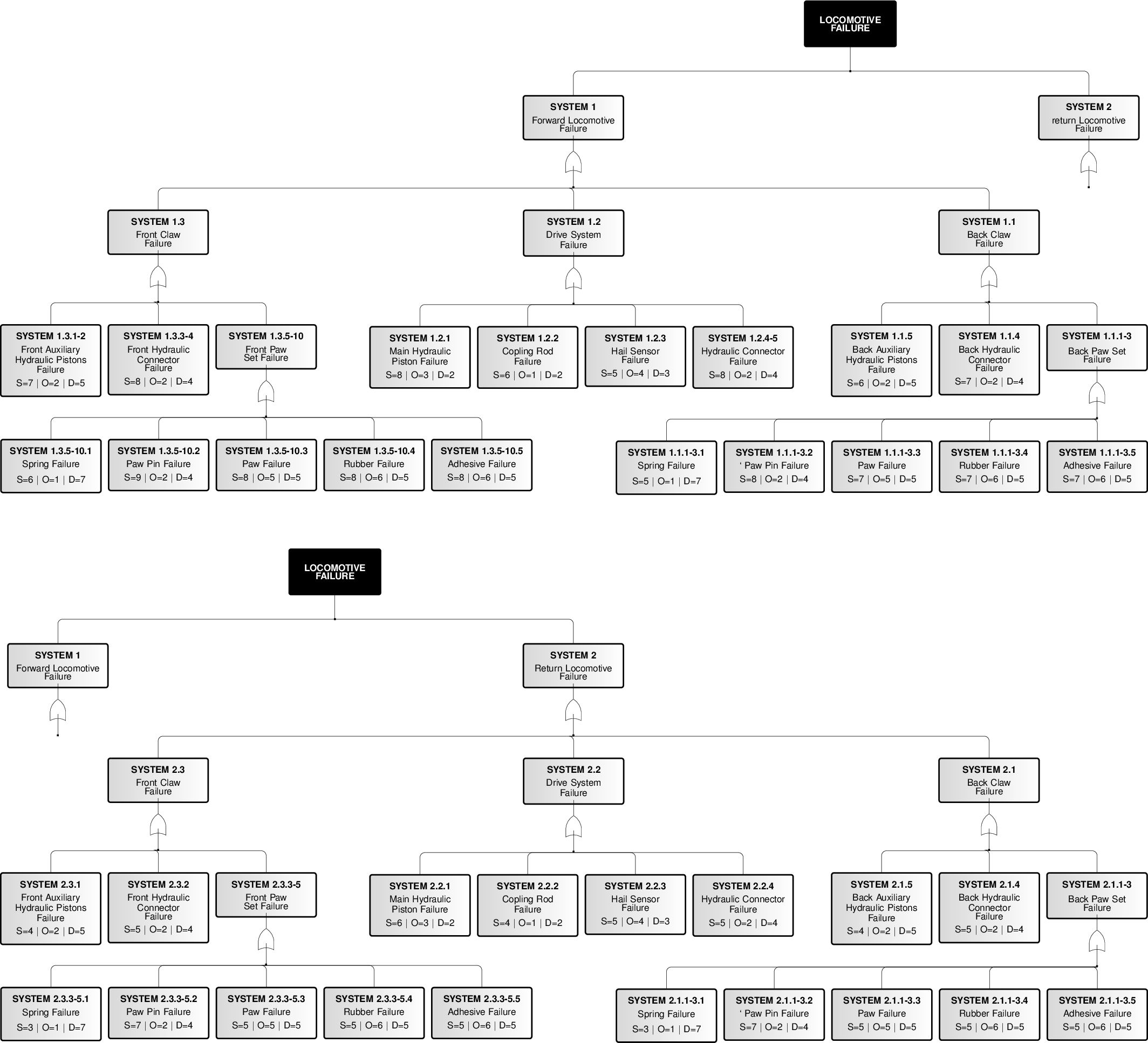

Supplement: S3 Fig — S = Severity, O = Occurrence, D = Detection. (TIF) [file pone.0255944.s003.tif]

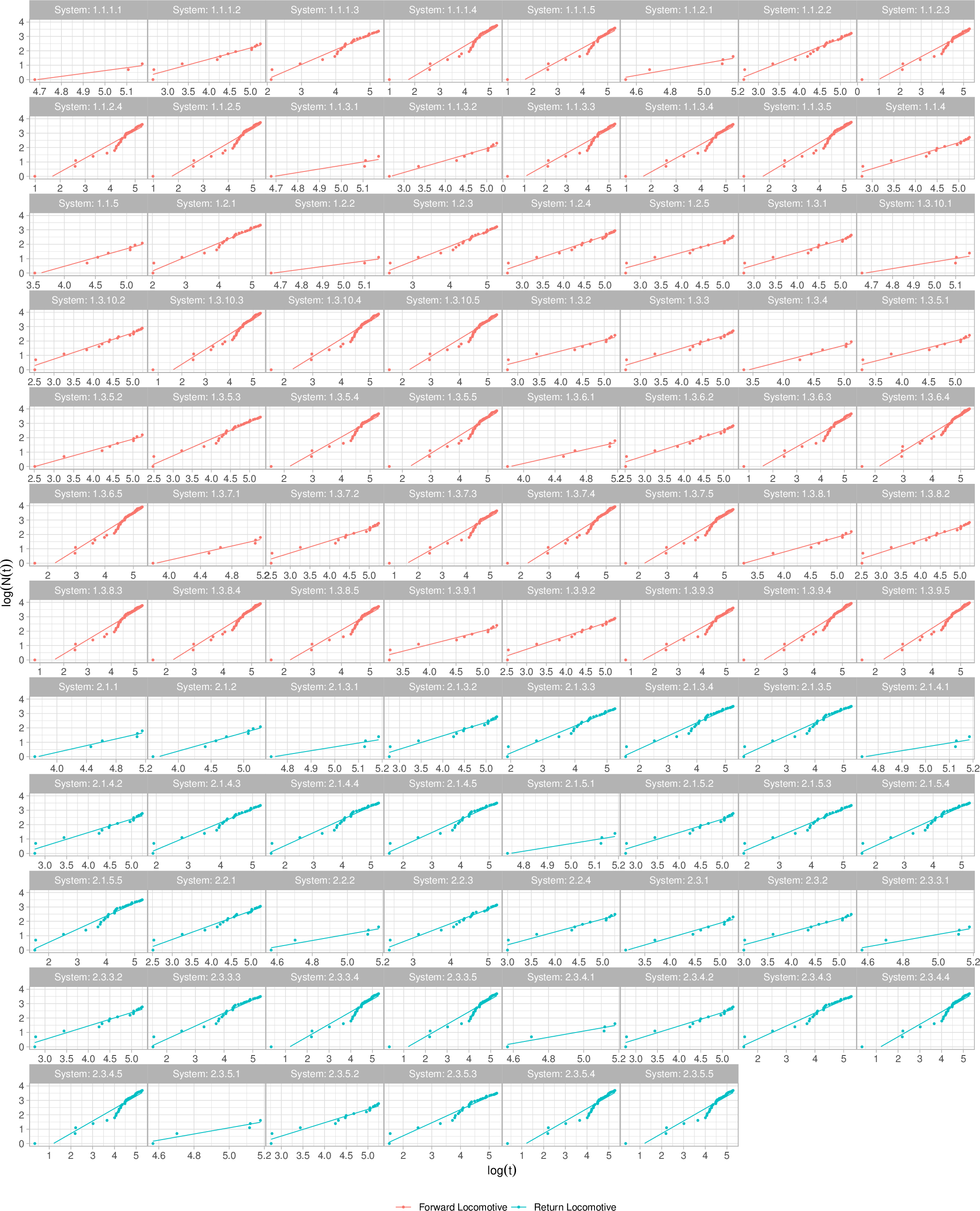

Supplement: S4 Fig — For the failure modes. (TIF) [file pone.0255944.s004.tif]

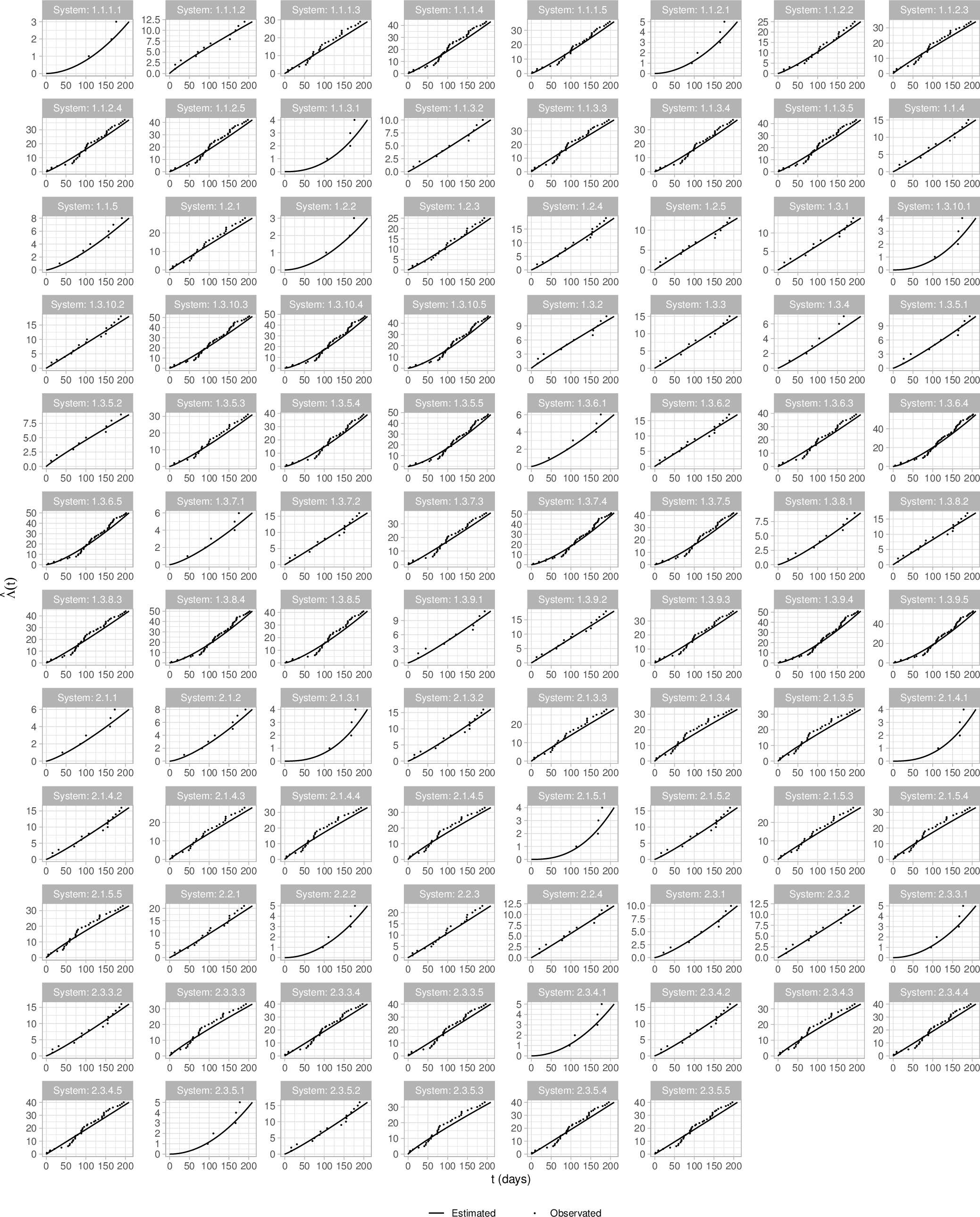

Supplement: S5 Fig — Number of observed and estimated failures per component. (TIF) [file pone.0255944.s005.tif]
