# Supplementary material for: Improved objective Bayesian estimator for a PLP model hierarchically represented subject to competing risks under minimal repair regime
Source: PLoS One. 2021 Aug 12;16(8):e0255944. doi: 10.1371/journal.pone.0255944 (PMC8360570; doi:10.1371/journal.pone.0255944)
Supplement: S1 Raw images — (PDF) [file pone.0255944.s007.pdf]

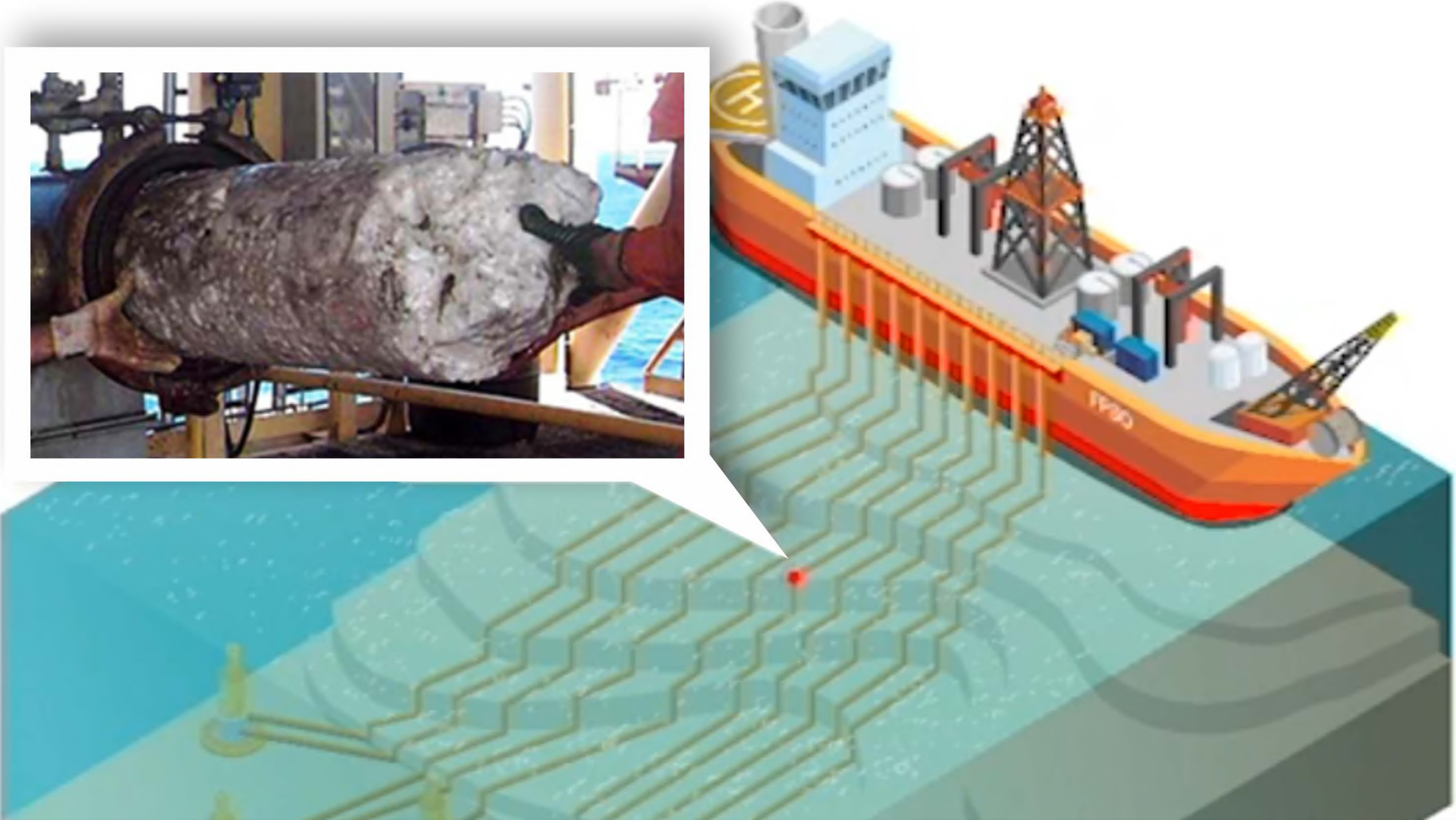

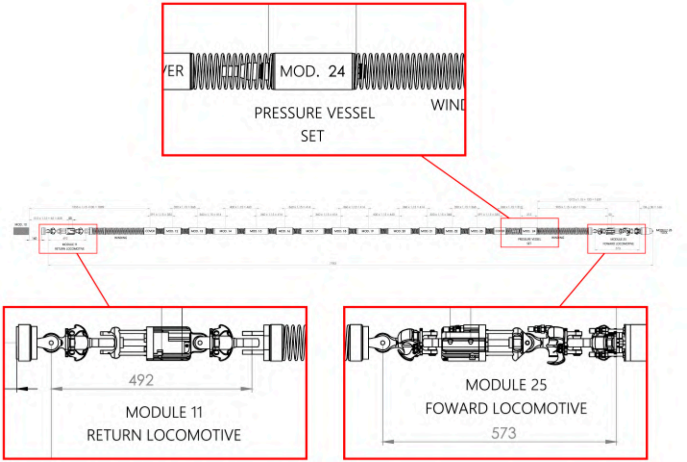

MOD. 24
PRESSURE VESSEL SET
WIND
492
MODULE 11
RETURN LOCOMOTIVE
MODULE 25
FOWARD LOCOMOTIVE
573

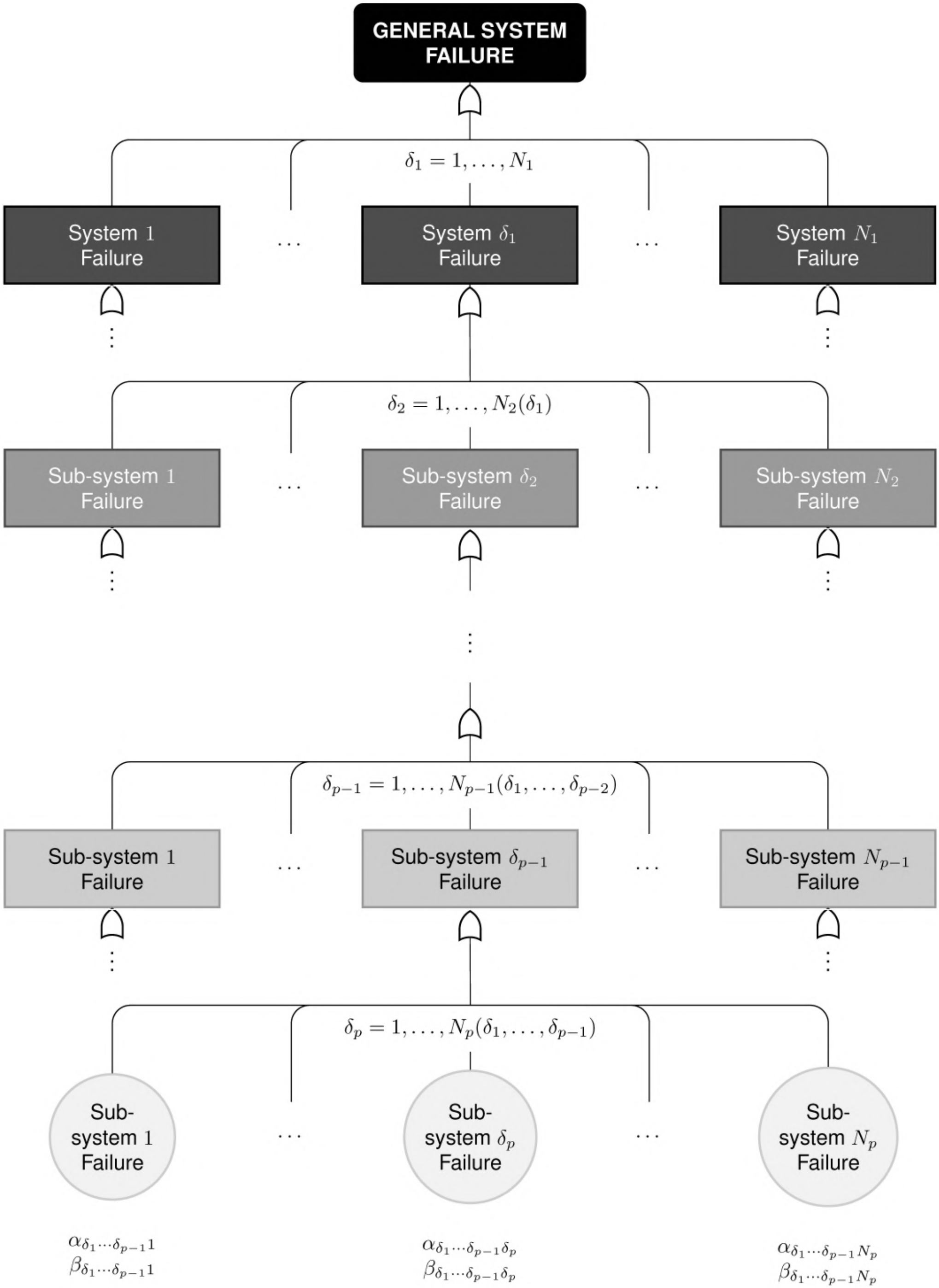
GENERAL SYSTEM FAILURE
$\delta_1 = 1, \dots, N_1$
System 1 Failure
System δ_1 Failure
System N_1 Failure
$\delta_2 = 1, \dots, N_2(\delta_1)$
Sub-system 1 Failure
Sub-system δ_2 Failure
Sub-system N_2 Failure
$\delta_{p-1} = 1, \dots, N_{p-1}(\delta_1, \dots, \delta_{p-2})$
Sub-system 1 Failure
Sub-system δ_{p-1} Failure
Sub-system N_{p-1} Failure
$\delta_p = 1, \dots, N_p(\delta_1, \dots, \delta_{p-1})$
Sub-system 1 Failure
Sub-system δ_p Failure
Sub-system N_p Failure
$\alpha_{\delta_1 \cdots \delta_{p-1} 1}$ $\beta_{\delta_1 \cdots \delta_{p-1} 1}$
$\alpha_{\delta_1 \cdots \delta_{p-1} \delta_p}$ $\beta_{\delta_1 \cdots \delta_{p-1} \delta_p}$
$\alpha_{\delta_1 \cdots \delta_{p-1} N_p}$ $\beta_{\delta_1 \cdots \delta_{p-1} N_p}$

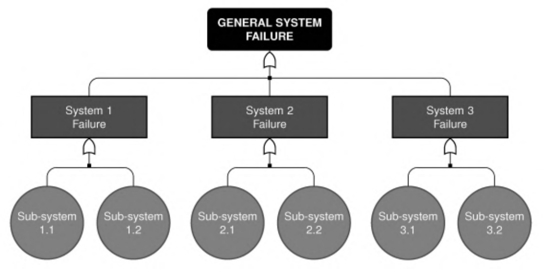
GENERAL SYSTEM FAILURE
System 1 Failure
System 2 Failure
System 3 Failure
Sub-system 1.1
Sub-system 1.2
Sub-system 2.1
Sub-system 2.2
Sub-system 3.1
Sub-system 3.2

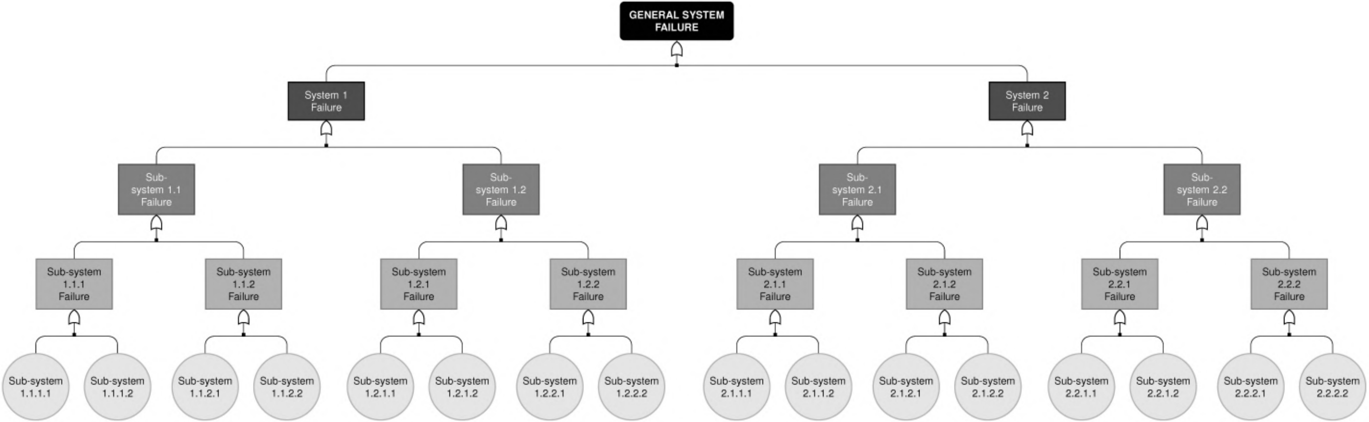
GENERAL SYSTEM FAILURE
System 1 Failure
System 2 Failure
Sub-system 1.1 Failure
Sub-system 1.2 Failure
Sub-system 2.1 Failure
Sub-system 2.2 Failure
Sub-system 1.1.1 Failure
Sub-system 1.1.2 Failure
Sub-system 1.2.1 Failure
Sub-system 1.2.2 Failure
Sub-system 2.1.1 Failure
Sub-system 2.1.2 Failure
Sub-system 2.2.1 Failure
Sub-system 2.2.2 Failure
Sub-system 1.1.1.1
Sub-system 1.1.1.2
Sub-system 1.1.2.1
Sub-system 1.1.2.2
Sub-system 1.2.1.1
Sub-system 1.2.1.2
Sub-system 1.2.2.1
Sub-system 1.2.2.2
Sub-system 2.1.1.1
Sub-system 2.1.1.2
Sub-system 2.1.2.1
Sub-system 2.1.2.2
Sub-system 2.2.1.1
Sub-system 2.2.1.2
Sub-system 2.2.2.1
Sub-system 2.2.2.2

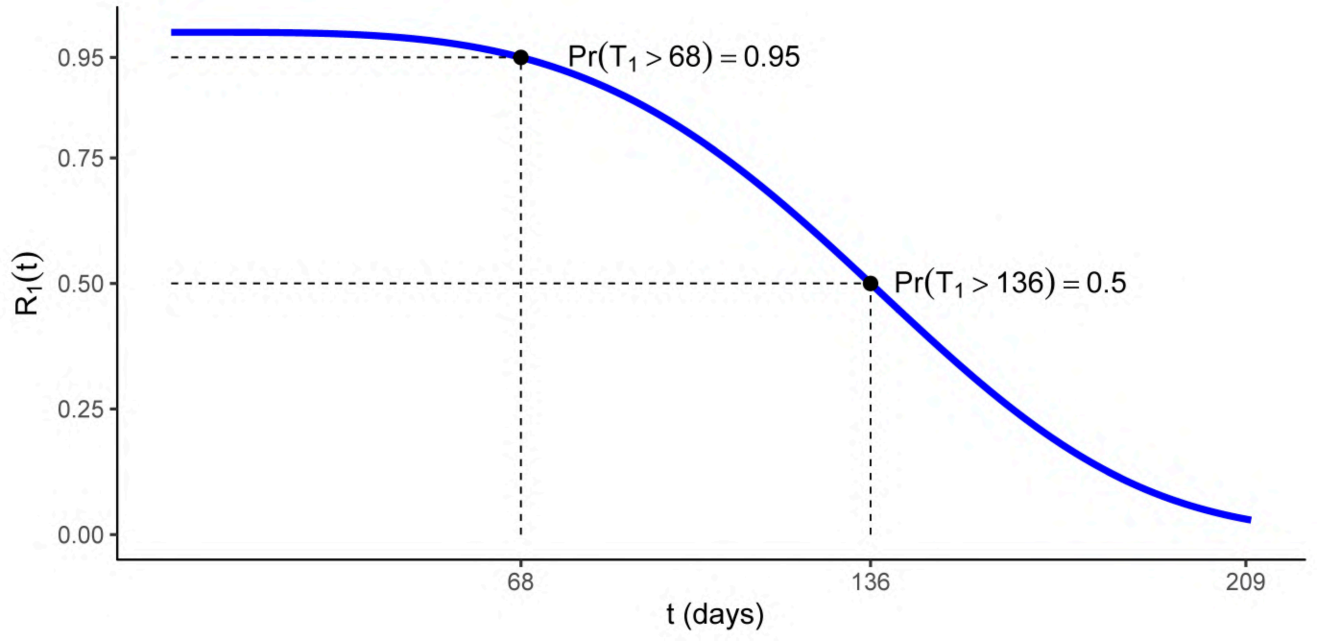

R1(t)
0.95
0.75
0.50
0.25
0.00
Pr(T1 > 68) = 0.95
Pr(T1 > 136) = 0.5
68
136
209
t (days)

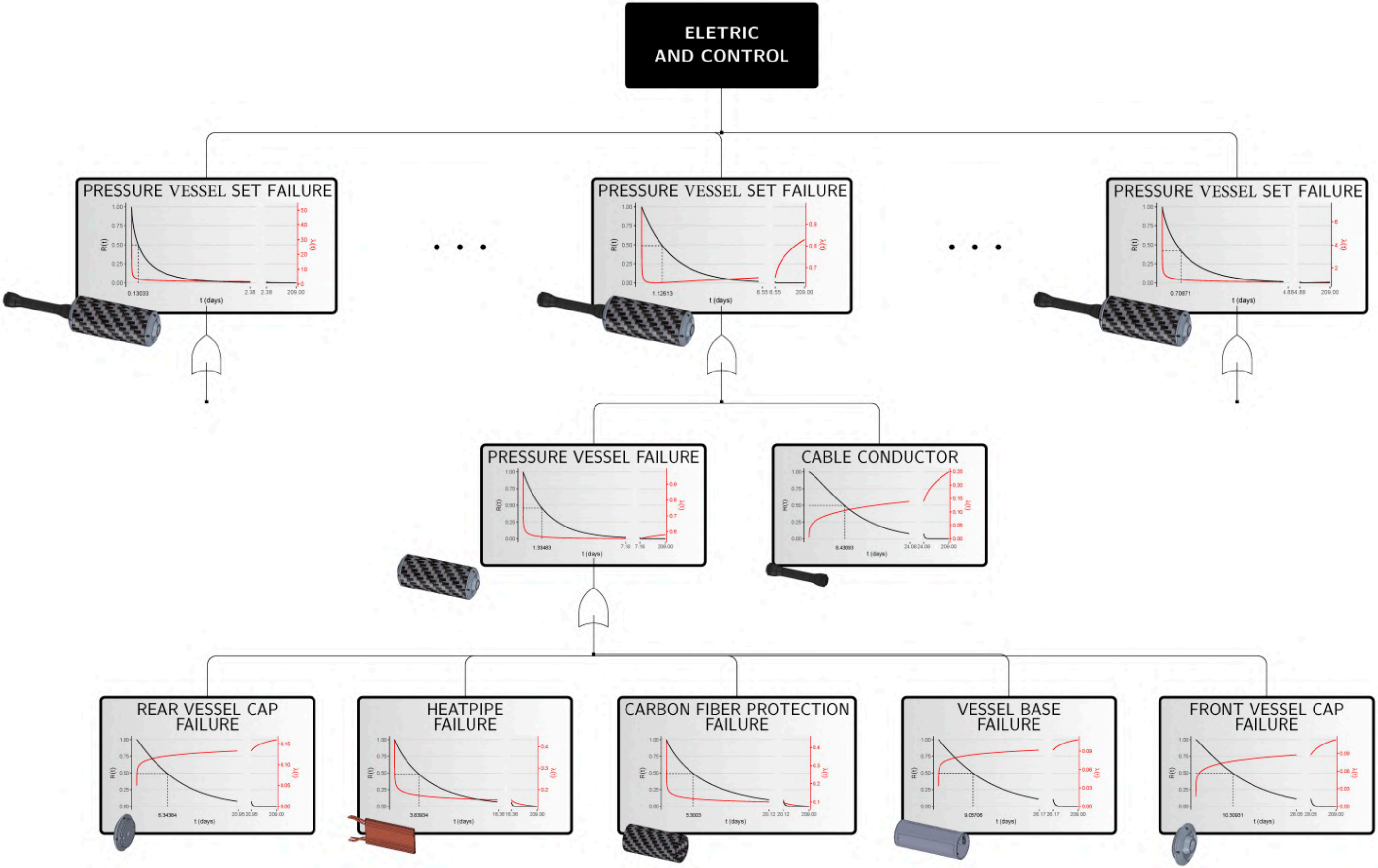
ELETRIC AND CONTROL
PRESSURE VESSEL SET FAILURE
PRESSURE VESSEL SET FAILURE
PRESSURE VESSEL SET FAILURE
PRESSURE VESSEL FAILURE
CABLE CONDUCTOR
REAR VESSEL CAP FAILURE
HEATPIPE FAILURE
CARBON FIBER PROTECTION FAILURE
VESSEL BASE FAILURE
FRONT VESSEL CAP FAILURE

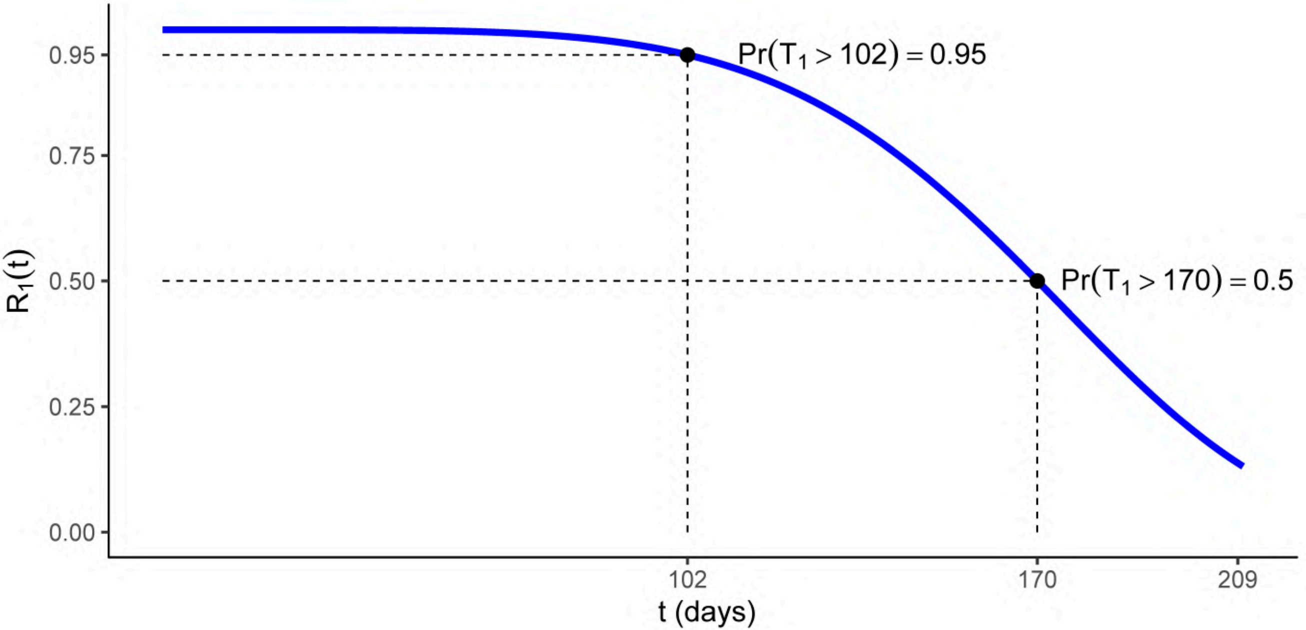

$Pr(T_1 > 102) = 0.95$
$Pr(T_1 > 170) = 0.5$
$R_1(t)$
0.95
0.75
0.50
0.25
0.00
102
170
209
t (days)

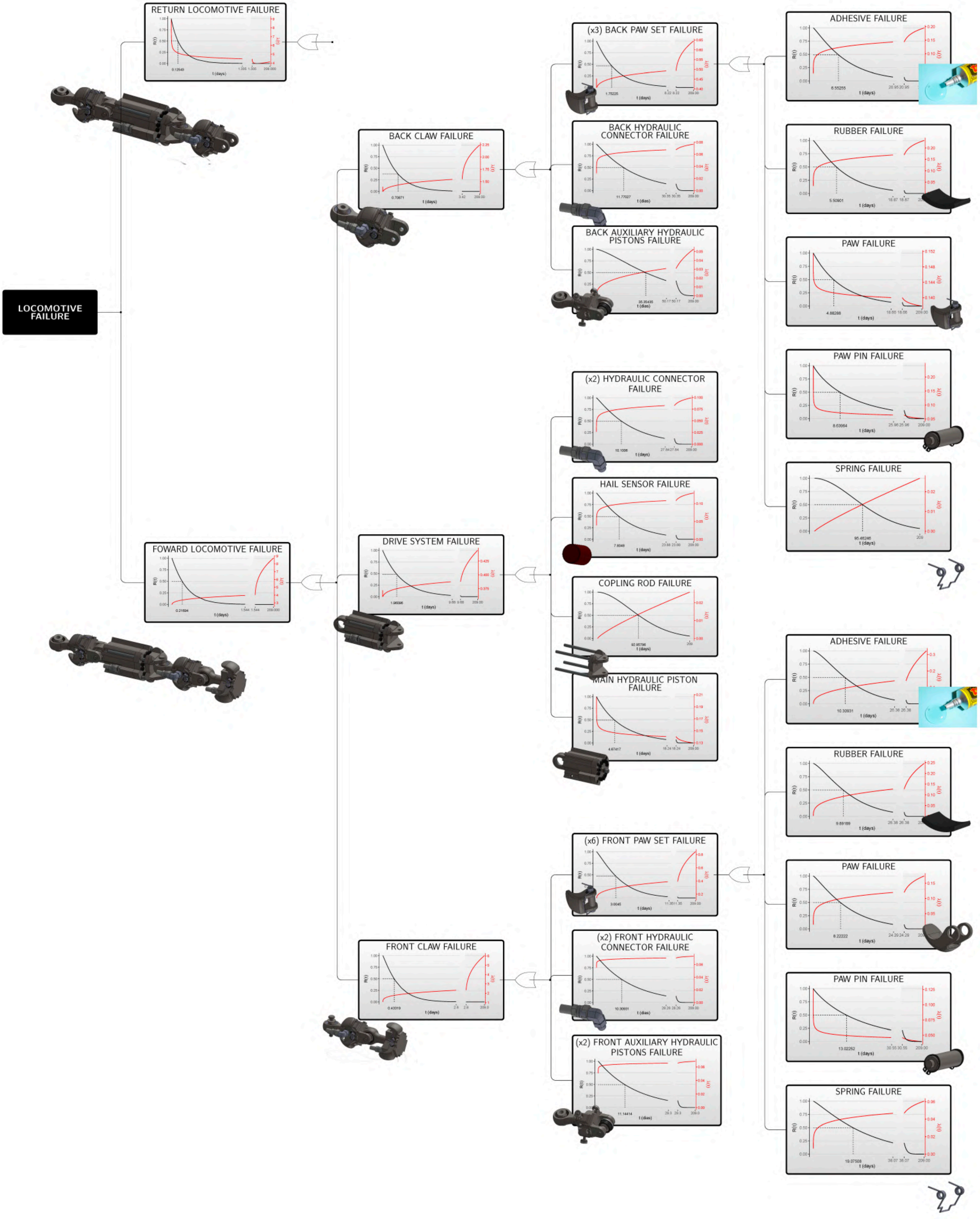
LOCOMOTIVE FAILURE
RETURN LOCOMOTIVE FAILURE
FOWARD LOCOMOTIVE FAILURE
BACK CLAW FAILURE
DRIVE SYSTEM FAILURE
FRONT CLAW FAILURE
(x3) BACK PAW SET FAILURE
BACK HYDRAULIC CONNECTOR FAILURE
BACK AUXILIARY HYDRAULIC PISTONS FAILURE
(x2) HYDRAULIC CONNECTOR FAILURE
HAIL SENSOR FAILURE
COPLING ROD FAILURE
MAIN HYDRAULIC PISTON FAILURE
(x6) FRONT PAW SET FAILURE
(x2) FRONT HYDRAULIC CONNECTOR FAILURE
(x2) FRONT AUXILIARY HYDRAULIC PISTONS FAILURE
ADHESIVE FAILURE
RUBBER FAILURE
PAW FAILURE
PAW PIN FAILURE
SPRING FAILURE
ADHESIVE FAILURE
RUBBER FAILURE
PAW FAILURE
PAW PIN FAILURE
SPRING FAILURE

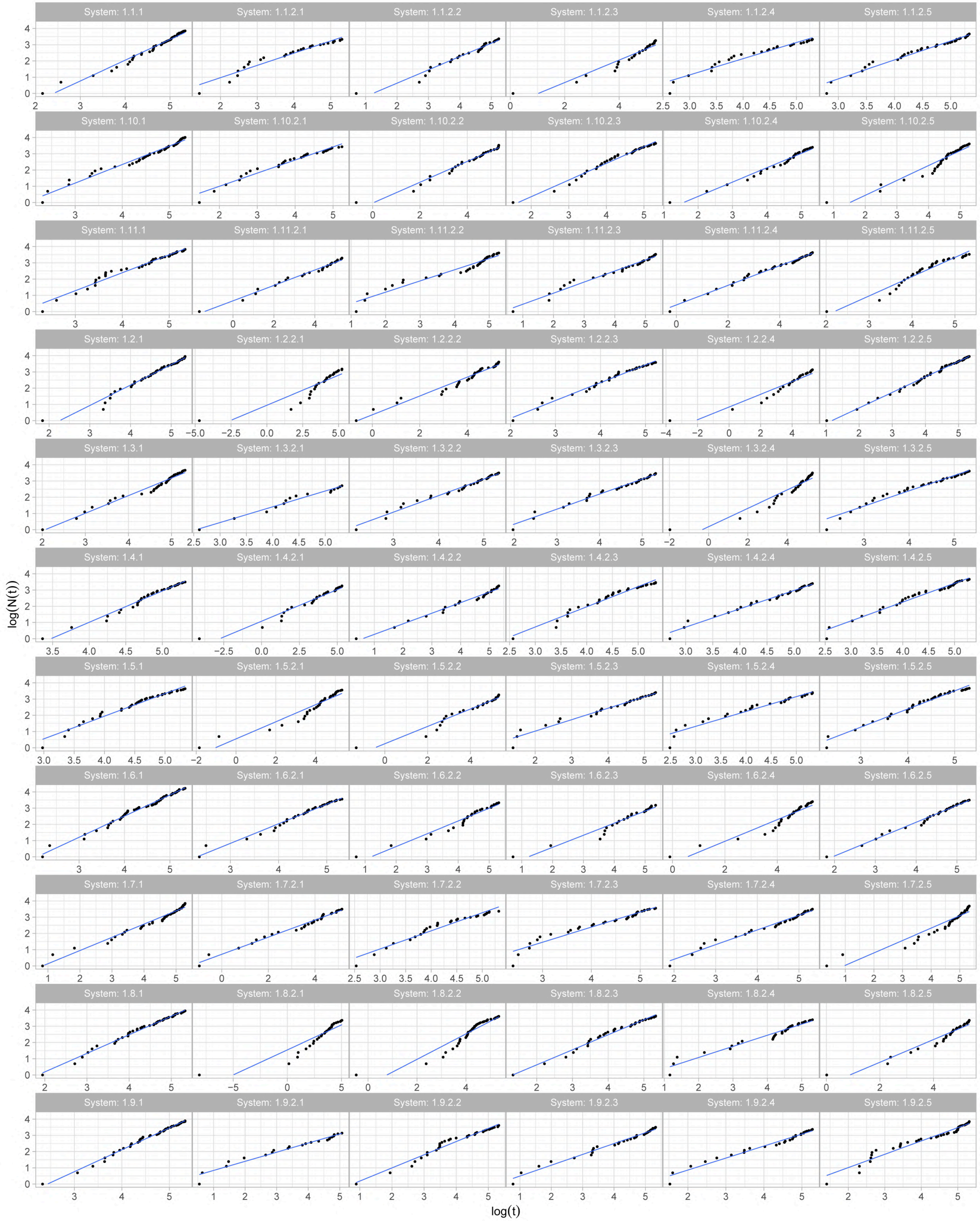
System: 1.1.1
System: 1.1.2.1
System: 1.1.2.2
System: 1.1.2.3
System: 1.1.2.4
System: 1.1.2.5
System: 1.10.1
System: 1.10.2.1
System: 1.10.2.2
System: 1.10.2.3
System: 1.10.2.4
System: 1.10.2.5
System: 1.11.1
System: 1.11.2.1
System: 1.11.2.2
System: 1.11.2.3
System: 1.11.2.4
System: 1.11.2.5
System: 1.2.1
System: 1.2.2.1
System: 1.2.2.2
System: 1.2.2.3
System: 1.2.2.4
System: 1.2.2.5
System: 1.3.1
System: 1.3.2.1
System: 1.3.2.2
System: 1.3.2.3
System: 1.3.2.4
System: 1.3.2.5
System: 1.4.1
System: 1.4.2.1
System: 1.4.2.2
System: 1.4.2.3
System: 1.4.2.4
System: 1.4.2.5
System: 1.5.1
System: 1.5.2.1
System: 1.5.2.2
System: 1.5.2.3
System: 1.5.2.4
System: 1.5.2.5
System: 1.6.1
System: 1.6.2.1
System: 1.6.2.2
System: 1.6.2.3
System: 1.6.2.4
System: 1.6.2.5
System: 1.7.1
System: 1.7.2.1
System: 1.7.2.2
System: 1.7.2.3
System: 1.7.2.4
System: 1.7.2.5
System: 1.8.1
System: 1.8.2.1
System: 1.8.2.2
System: 1.8.2.3
System: 1.8.2.4
System: 1.8.2.5
System: 1.9.1
System: 1.9.2.1
System: 1.9.2.2
System: 1.9.2.3
System: 1.9.2.4
System: 1.9.2.5
log(N(t))
log(t)

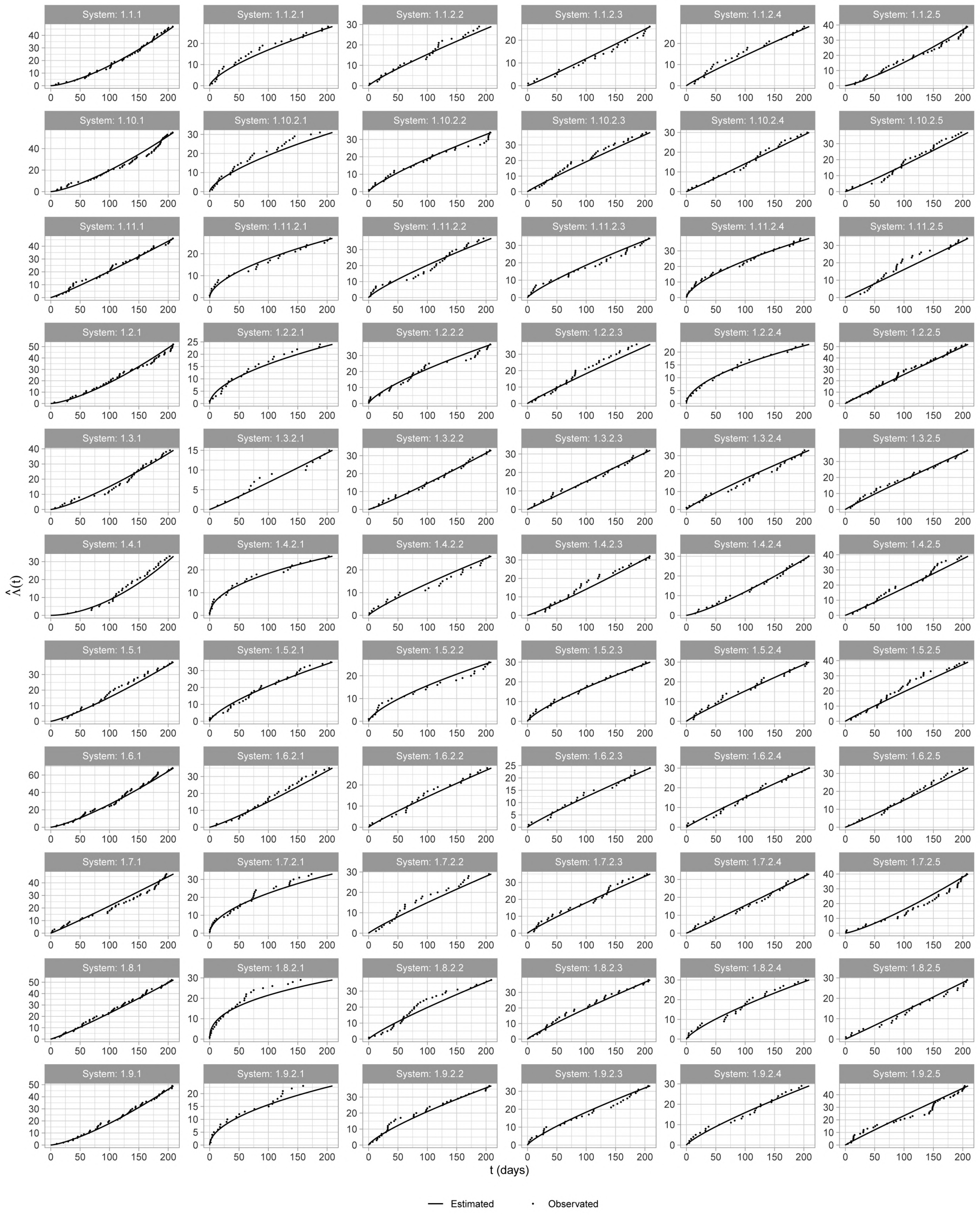

System: 1.1.1
System: 1.1.2.1
System: 1.1.2.2
System: 1.1.2.3
System: 1.1.2.4
System: 1.1.2.5
System: 1.10.1
System: 1.10.2.1
System: 1.10.2.2
System: 1.10.2.3
System: 1.10.2.4
System: 1.10.2.5
System: 1.11.1
System: 1.11.2.1
System: 1.11.2.2
System: 1.11.2.3
System: 1.11.2.4
System: 1.11.2.5
System: 1.2.1
System: 1.2.2.1
System: 1.2.2.2
System: 1.2.2.3
System: 1.2.2.4
System: 1.2.2.5
System: 1.3.1
System: 1.3.2.1
System: 1.3.2.2
System: 1.3.2.3
System: 1.3.2.4
System: 1.3.2.5
System: 1.4.1
System: 1.4.2.1
System: 1.4.2.2
System: 1.4.2.3
System: 1.4.2.4
System: 1.4.2.5
System: 1.5.1
System: 1.5.2.1
System: 1.5.2.2
System: 1.5.2.3
System: 1.5.2.4
System: 1.5.2.5
System: 1.6.1
System: 1.6.2.1
System: 1.6.2.2
System: 1.6.2.3
System: 1.6.2.4
System: 1.6.2.5
System: 1.7.1
System: 1.7.2.1
System: 1.7.2.2
System: 1.7.2.3
System: 1.7.2.4
System: 1.7.2.5
System: 1.8.1
System: 1.8.2.1
System: 1.8.2.2
System: 1.8.2.3
System: 1.8.2.4
System: 1.8.2.5
System: 1.9.1
System: 1.9.2.1
System: 1.9.2.2
System: 1.9.2.3
System: 1.9.2.4
System: 1.9.2.5
$\hat{\Lambda}(t)$
t (days)
Estimated
Observed

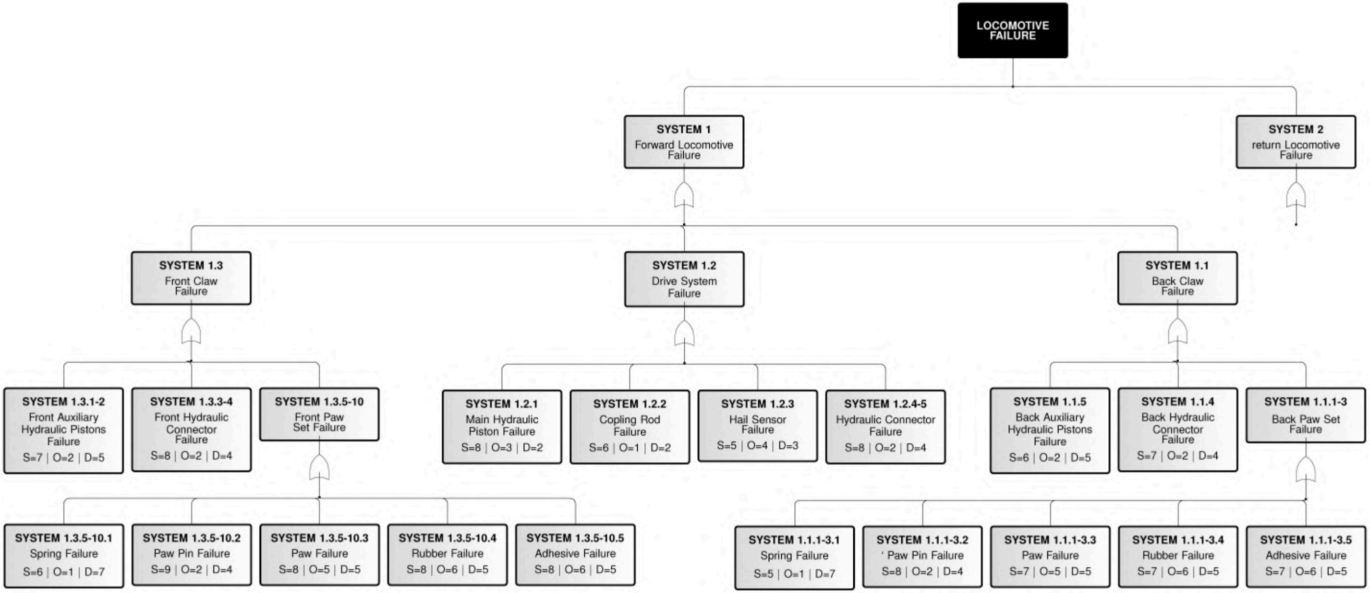
LOCOMOTIVE FAILURE
SYSTEM 1
Forward Locomotive Failure
SYSTEM 2
return Locomotive Failure
SYSTEM 1.3
Front Claw Failure
SYSTEM 1.2
Drive System Failure
SYSTEM 1.1
Back Claw Failure
SYSTEM 1.3.1-2
Front Auxiliary Hydraulic Pistons Failure
S=7 | O=2 | D=5
SYSTEM 1.3.3-4
Front Hydraulic Connector Failure
S=8 | O=2 | D=4
SYSTEM 1.3.5-10
Front Paw Set Failure
SYSTEM 1.2.1
Main Hydraulic Piston Failure
S=8 | O=3 | D=2
SYSTEM 1.2.2
Copling Rod Failure
S=6 | O=1 | D=2
SYSTEM 1.2.3
Hail Sensor Failure
S=5 | O=4 | D=3
SYSTEM 1.2.4-5
Hydraulic Connector Failure
S=8 | O=2 | D=4
SYSTEM 1.1.5
Back Auxiliary Hydraulic Pistons Failure
S=6 | O=2 | D=5
SYSTEM 1.1.4
Back Hydraulic Connector Failure
S=7 | O=2 | D=4
SYSTEM 1.1.1-3
Back Paw Set Failure
SYSTEM 1.3.5-10.1
Spring Failure
S=6 | O=1 | D=7
SYSTEM 1.3.5-10.2
Paw Pin Failure
S=9 | O=2 | D=4
SYSTEM 1.3.5-10.3
Paw Failure
S=8 | O=5 | D=5
SYSTEM 1.3.5-10.4
Rubber Failure
S=8 | O=6 | D=5
SYSTEM 1.3.5-10.5
Adhesive Failure
S=8 | O=6 | D=5
SYSTEM 1.1.1-3.1
Spring Failure
S=5 | O=1 | D=7
SYSTEM 1.1.1-3.2
' Paw Pin Failure
S=8 | O=2 | D=4
SYSTEM 1.1.1-3.3
Paw Failure
S=7 | O=5 | D=5
SYSTEM 1.1.1-3.4
Rubber Failure
S=7 | O=6 | D=5
SYSTEM 1.1.1-3.5
Adhesive Failure
S=7 | O=6 | D=5

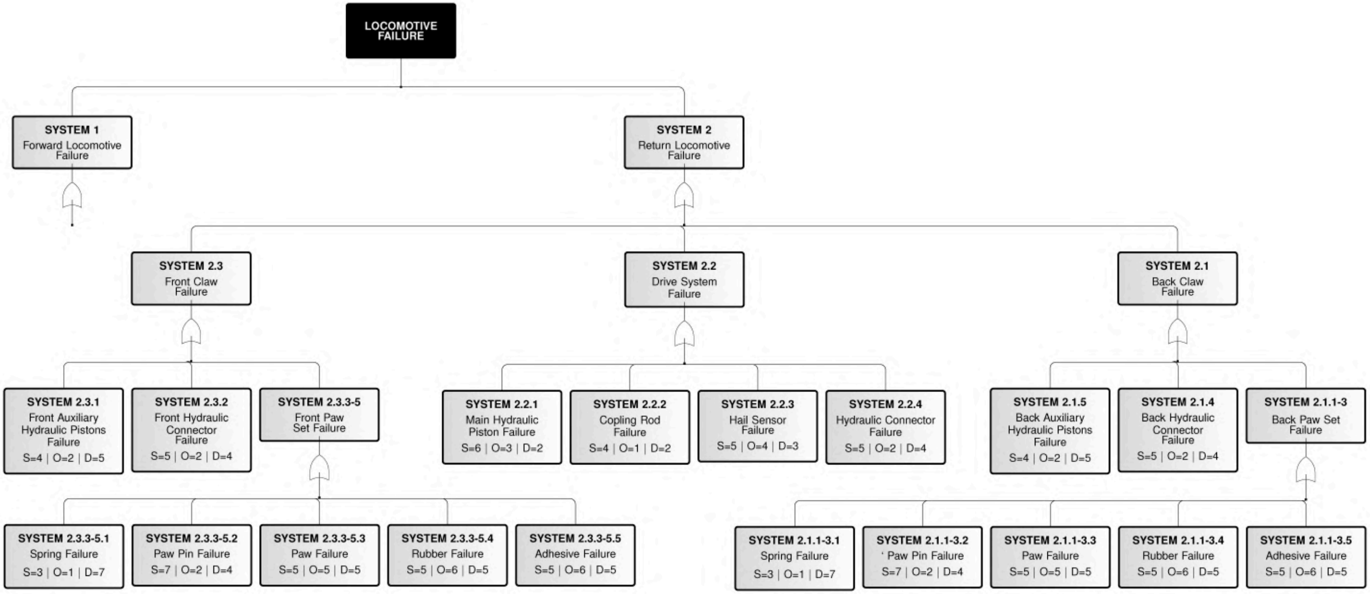
LOCOMOTIVE FAILURE
SYSTEM 1
Forward Locomotive Failure
SYSTEM 2
Return Locomotive Failure
SYSTEM 2.3
Front Claw Failure
SYSTEM 2.2
Drive System Failure
SYSTEM 2.1
Back Claw Failure
SYSTEM 2.3.1
Front Auxiliary Hydraulic Pistons Failure
S=4 | O=2 | D=5
SYSTEM 2.3.2
Front Hydraulic Connector Failure
S=5 | O=2 | D=4
SYSTEM 2.3.3-5
Front Paw Set Failure
SYSTEM 2.2.1
Main Hydraulic Piston Failure
S=6 | O=3 | D=2
SYSTEM 2.2.2
Copling Rod Failure
S=4 | O=1 | D=2
SYSTEM 2.2.3
Hail Sensor Failure
S=5 | O=4 | D=3
SYSTEM 2.2.4
Hydraulic Connector Failure
S=5 | O=2 | D=4
SYSTEM 2.1.5
Back Auxiliary Hydraulic Pistons Failure
S=4 | O=2 | D=5
SYSTEM 2.1.4
Back Hydraulic Connector Failure
S=5 | O=2 | D=4
SYSTEM 2.1.1-3
Back Paw Set Failure
SYSTEM 2.3.3-5.1
Spring Failure
S=3 | O=1 | D=7
SYSTEM 2.3.3-5.2
Paw Pin Failure
S=7 | O=2 | D=4
SYSTEM 2.3.3-5.3
Paw Failure
S=5 | O=5 | D=5
SYSTEM 2.3.3-5.4
Rubber Failure
S=5 | O=6 | D=5
SYSTEM 2.3.3-5.5
Adhesive Failure
S=5 | O=6 | D=5
SYSTEM 2.1.1-3.1
Spring Failure
S=3 | O=1 | D=7
SYSTEM 2.1.1-3.2
' Paw Pin Failure
S=7 | O=2 | D=4
SYSTEM 2.1.1-3.3
Paw Failure
S=5 | O=5 | D=5
SYSTEM 2.1.1-3.4
Rubber Failure
S=5 | O=6 | D=5
SYSTEM 2.1.1-3.5
Adhesive Failure
S=5 | O=6 | D=5

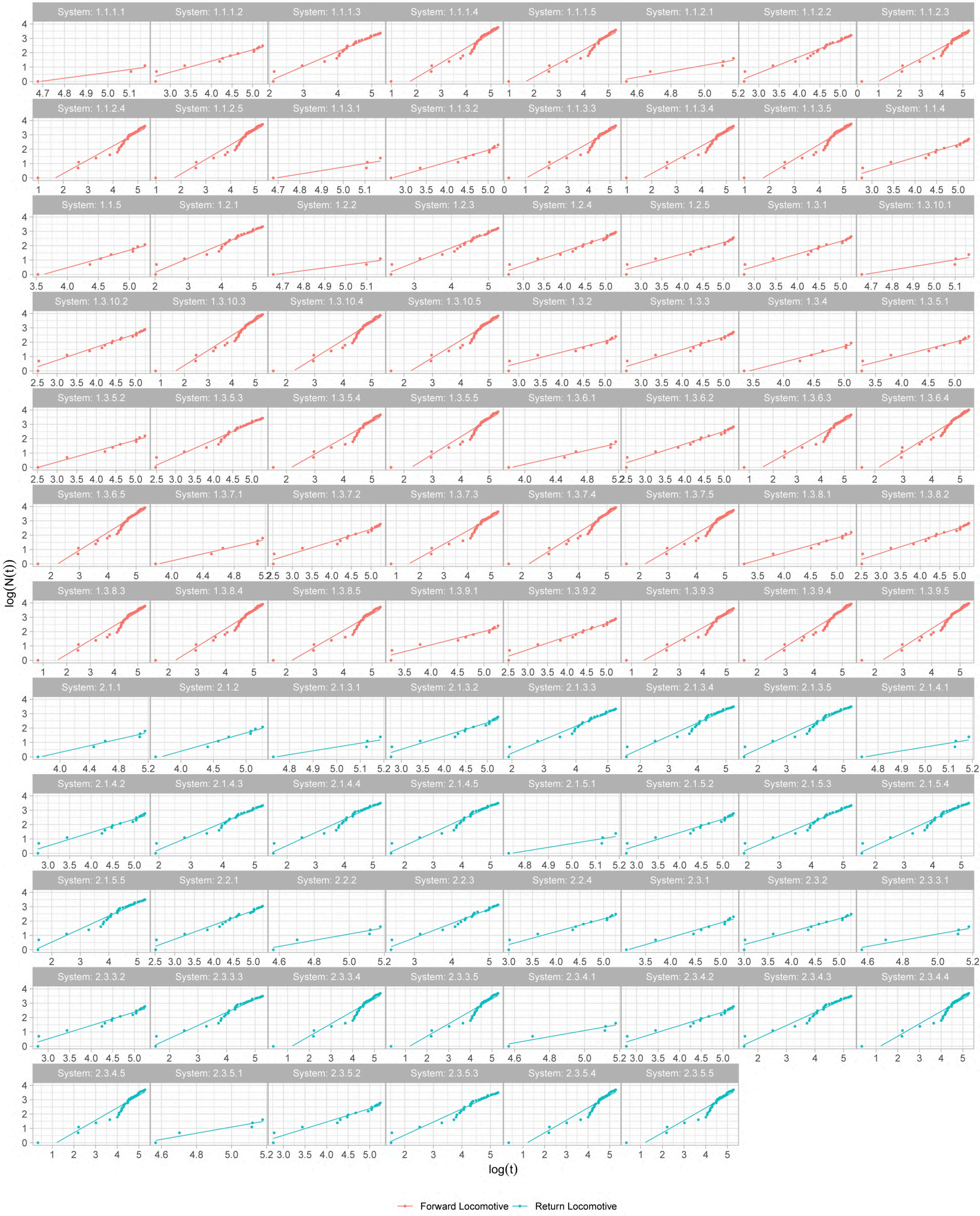
System: 1.1.1.1
System: 1.1.1.2
System: 1.1.1.3
System: 1.1.1.4
System: 1.1.1.5
System: 1.1.2.1
System: 1.1.2.2
System: 1.1.2.3
System: 1.1.2.4
System: 1.1.2.5
System: 1.1.3.1
System: 1.1.3.2
System: 1.1.3.3
System: 1.1.3.4
System: 1.1.3.5
System: 1.1.4
System: 1.1.5
System: 1.2.1
System: 1.2.2
System: 1.2.3
System: 1.2.4
System: 1.2.5
System: 1.3.1
System: 1.3.10.1
System: 1.3.10.2
System: 1.3.10.3
System: 1.3.10.4
System: 1.3.10.5
System: 1.3.2
System: 1.3.3
System: 1.3.4
System: 1.3.5.1
System: 1.3.5.2
System: 1.3.5.3
System: 1.3.5.4
System: 1.3.5.5
System: 1.3.6.1
System: 1.3.6.2
System: 1.3.6.3
System: 1.3.6.4
System: 1.3.6.5
System: 1.3.7.1
System: 1.3.7.2
System: 1.3.7.3
System: 1.3.7.4
System: 1.3.7.5
System: 1.3.8.1
System: 1.3.8.2
System: 1.3.8.3
System: 1.3.8.4
System: 1.3.8.5
System: 1.3.9.1
System: 1.3.9.2
System: 1.3.9.3
System: 1.3.9.4
System: 1.3.9.5
System: 2.1.1
System: 2.1.2
System: 2.1.3.1
System: 2.1.3.2
System: 2.1.3.3
System: 2.1.3.4
System: 2.1.3.5
System: 2.1.4.1
System: 2.1.4.2
System: 2.1.4.3
System: 2.1.4.4
System: 2.1.4.5
System: 2.1.5.1
System: 2.1.5.2
System: 2.1.5.3
System: 2.1.5.4
System: 2.1.5.5
System: 2.2.1
System: 2.2.2
System: 2.2.3
System: 2.2.4
System: 2.3.1
System: 2.3.2
System: 2.3.3.1
System: 2.3.3.2
System: 2.3.3.3
System: 2.3.3.4
System: 2.3.3.5
System: 2.3.4.1
System: 2.3.4.2
System: 2.3.4.3
System: 2.3.4.4
System: 2.3.4.5
System: 2.3.5.1
System: 2.3.5.2
System: 2.3.5.3
System: 2.3.5.4
System: 2.3.5.5
log(N(t))
log(t)
Forward Locomotive
Return Locomotive

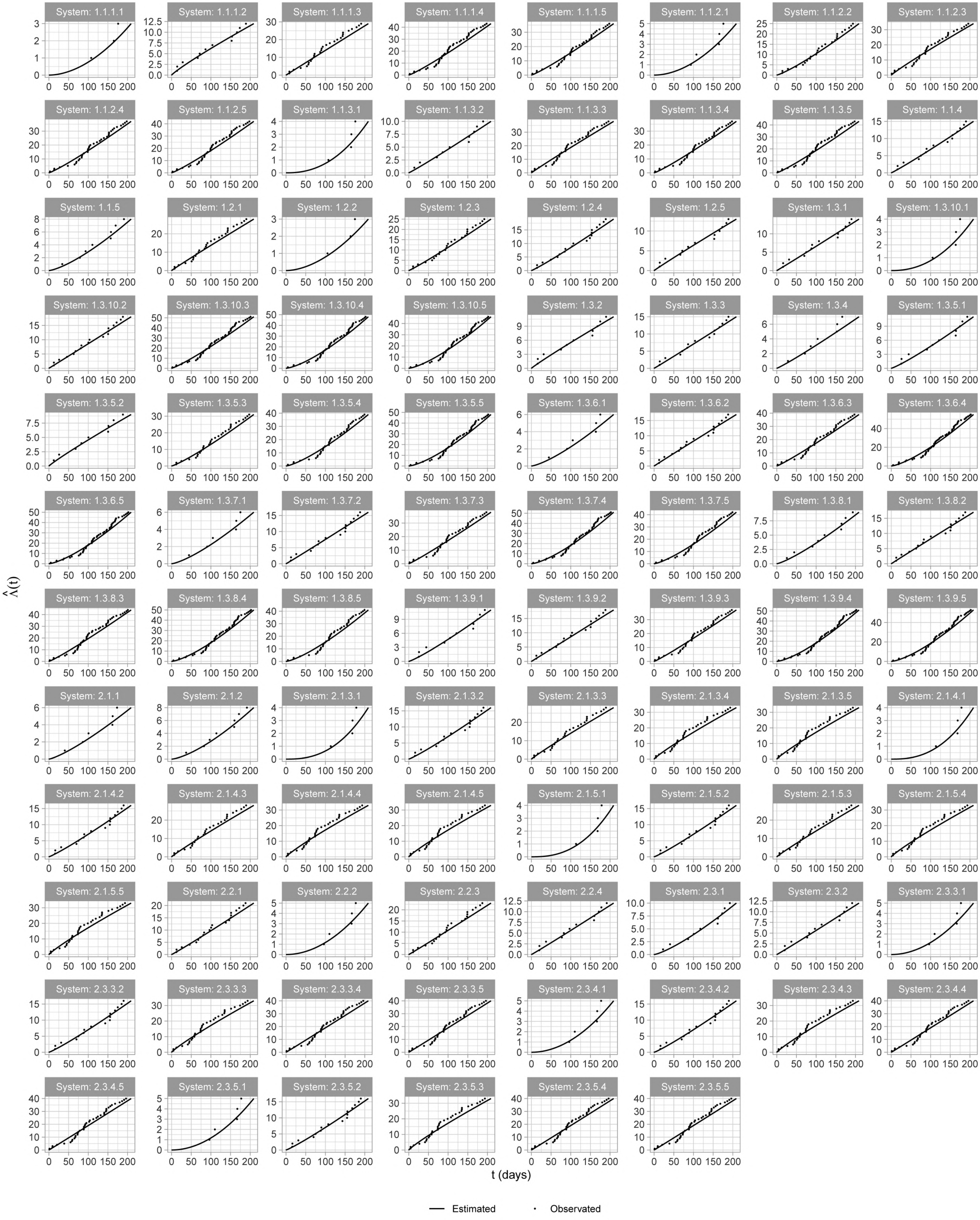

System: 1.1.1.1
System: 1.1.1.2
System: 1.1.1.3
System: 1.1.1.4
System: 1.1.1.5
System: 1.1.2.1
System: 1.1.2.2
System: 1.1.2.3
System: 1.1.2.4
System: 1.1.2.5
System: 1.1.3.1
System: 1.1.3.2
System: 1.1.3.3
System: 1.1.3.4
System: 1.1.3.5
System: 1.1.4
System: 1.1.5
System: 1.2.1
System: 1.2.2
System: 1.2.3
System: 1.2.4
System: 1.2.5
System: 1.3.1
System: 1.3.10.1
System: 1.3.10.2
System: 1.3.10.3
System: 1.3.10.4
System: 1.3.10.5
System: 1.3.2
System: 1.3.3
System: 1.3.4
System: 1.3.5.1
System: 1.3.5.2
System: 1.3.5.3
System: 1.3.5.4
System: 1.3.5.5
System: 1.3.6.1
System: 1.3.6.2
System: 1.3.6.3
System: 1.3.6.4
System: 1.3.6.5
System: 1.3.7.1
System: 1.3.7.2
System: 1.3.7.3
System: 1.3.7.4
System: 1.3.7.5
System: 1.3.8.1
System: 1.3.8.2
System: 1.3.8.3
System: 1.3.8.4
System: 1.3.8.5
System: 1.3.9.1
System: 1.3.9.2
System: 1.3.9.3
System: 1.3.9.4
System: 1.3.9.5
System: 2.1.1
System: 2.1.2
System: 2.1.3.1
System: 2.1.3.2
System: 2.1.3.3
System: 2.1.3.4
System: 2.1.3.5
System: 2.1.4.1
System: 2.1.4.2
System: 2.1.4.3
System: 2.1.4.4
System: 2.1.4.5
System: 2.1.5.1
System: 2.1.5.2
System: 2.1.5.3
System: 2.1.5.4
System: 2.1.5.5
System: 2.2.1
System: 2.2.2
System: 2.2.3
System: 2.2.4
System: 2.3.1
System: 2.3.2
System: 2.3.3.1
System: 2.3.3.2
System: 2.3.3.3
System: 2.3.3.4
System: 2.3.3.5
System: 2.3.4.1
System: 2.3.4.2
System: 2.3.4.3
System: 2.3.4.4
System: 2.3.4.5
System: 2.3.5.1
System: 2.3.5.2
System: 2.3.5.3
System: 2.3.5.4
System: 2.3.5.5
$\hat{\Lambda}(t)$
t (days)
Estimated
Observed
